# Optimizing riociguat therapy for pulmonary hypertension: A systematic review and meta-analysis of dose variability, safety, and efficacy

**DOI:** 10.1097/MD.0000000000046007

**Published:** 2026-04-24

**Authors:** Muhammad Aqib Faizan, Tooba Rehman, Hurais Malik, Muhammad Hudaib, Muhammad Abdullah Tahir, Gunvi Ohri, Saher Bano, Laveeza Fatima, Syed Hassan Ahmed, Ayat ul Karam, Zuhair Ahmed, Abdul Rehman, Samra Rabbani, Mrunalini Dandamudi, Mohammed Mahmmoud Fadelallah Eljack, Zainab Humayun

**Affiliations:** a Department of Medicine, Gomal Medical College, Khyber Medical University, Pakistan; b Department of Medicine, Fazaia Ruth Pfau Medical College, Karachi, Pakistan; c Department of Medicine, Dr. Sampurnanand Medical College, Jodhpur, India; d Department of Medicine, Ayub Medical College, Abbottabad, Pakistan; e Department of Medicine, Allama Iqbal Medical College, Lahore, Pakistan; f Department of Medicine, Ruth KM Pfau Civil Hospital, Karachi, Pakistan; g Department of Medicine, Dow University of Health Sciences, Karachi, Pakistan; h Department of Medicine, Montefiore Einstein Hospital, New York, NY; i Department of Community Medicine, University of Bakht Alruda, Ed Dueim, Sudan; j Department of Medicine, University of Missouri, Kansas City, MO.

**Keywords:** dose variability, efficacy, meta-analysis, pulmonary hypertension, randomized controlled trials, riociguat, safety, systematic review

## Abstract

**Background:**

Pulmonary hypertension (PH) is a progressive, life-threatening condition characterized by elevated pulmonary arterial pressure, often culminating in right heart failure. Despite existing therapies, optimizing treatment efficacy and safety remains a clinical challenge. Riociguat, a soluble guanylate cyclase stimulator, has shown promise in managing PH, particularly in pulmonary arterial hypertension (PAH) and chronic thromboembolic pulmonary hypertension (CTEPH). This meta-analysis aims to evaluate the optimal dosing of riociguat to maximize therapeutic benefits while minimizing adverse effects.

**Methods:**

This systematic review and meta-analysis followed PRISMA guidelines. A comprehensive search of PubMed, Cochrane Central, Embase, and Google Scholar was conducted to identify randomized controlled trials (RCTs) comparing riociguat with placebo in adult PH patients. Eligible studies included double-arm RCTs with riociguat dosages ranging from 0.5 to 2.5 mg. Primary outcomes included changes in 6-minute walk distance (6-MWD), mean pulmonary arterial pressure (mPAP), pulmonary vascular resistance (PVR), and NT-proBNP levels. Secondary outcomes were adverse events and clinical worsening. Study quality was assessed using the Cochrane risk of bias tool. Statistical analysis used RevMan 5.4 with a random-effects model. Heterogeneity was evaluated using *χ*^2^ and *I*^2^ tests, and meta-regression assessed the impact of age and BMI.

**Results:**

Ten RCTs involving 1109 PH patients were included. Riociguat significantly improved 6-MWD (MD: 27.23 m; 95% CI: 8.28–46.18; *P* = .005), reduced mPAP (MD: −2.61 mm Hg; 95% CI: −4.60 to −0.63; *P* = .01), lowered PVR (MD: −90.27 dynes·s·cm^−5^; 95% CI: −119.30 to −61.23; *P* < .00001), and decreased NT-proBNP levels (MD: −256.77 pg/mL; 95% CI: −491.99 to −21.55; *P* = .03). Meta-regression showed age and BMI significantly influenced treatment effects.

**Conclusions:**

Riociguat demonstrates dose-dependent efficacy in improving functional and hemodynamic parameters in PH, with the 2.5 mg thrice-daily dose showing consistent benefit. BMI impacted functional outcomes but not hemodynamic measures. These findings support tailored dosing strategies for optimized patient outcomes. Further research is needed to confirm dose-response effects and address residual heterogeneity.

## 1. Introduction

Pulmonary hypertension (PH), a debilitating condition affecting millions worldwide, poses a significant challenge in pulmonary medicine and is linked with substantial morbidity and mortality. It is a complex and progressive condition characterized by elevated mean pulmonary arterial pressure (m PAP) equal to or >25 mm Hg at rest, leading to right heart failure and, ultimately, premature mortality.^[1]^ PH is a serious global health challenge affecting approximately 1% of the world’s population,^[2]^ with an even higher prevalence among patients with chronic heart and lung diseases. The global burden of PH is growing, especially in association with chronic conditions like chronic obstructive pulmonary disease (COPD) and heart failure. For instance, COPD-related PH affects about 39% of COPD patients, with a significant regional variation, highest in Africa (64%) and lowest in Europe (30%).^[3]^ Similarly, heart failure-induced PH shows substantial variability in prevalence across regions, with Europe having the highest rate (66%) compared to North America (17%).^[4]^

PH encompasses various subtypes, which have been categorized by the WHO into 5 classifications: pulmonary arterial hypertension (PAH, group 1), PH due to left heart disease (PH-LHD, group 2), PH due to lung disease (LD-PH, group 3), chronic thromboembolic pulmonary hypertension (CTEPH, group 4), and PH with unclear or multifactorial mechanisms (group 5).^[2]^ Traditional treatments, such as endothelin receptor antagonists (ERAs) and phosphodiesterase-5 (PDE5) inhibitors, often fail to fully control disease progression due to their limited impact on certain pathophysiological mechanisms. For example, PDE5 inhibitors rely on endogenous nitric oxide (NO), which is often deficient in PH patients, thus limiting their effectiveness.^[5]^ CTPH has a standard curative procedure called Pulmonary endarterectomy (PEA)^[6]^ However, up to 40% of patients with CTEPH are considered technically inoperable, while up to 51% of patients develop persistent/recurrent PH after undergoing PEA.^[7]^ Despite advancements in therapeutic modalities, the exigency for comprehensive evaluations of emerging treatments persists to refine clinical management strategies and enhance patient outcomes.

Recent advances in the pharmacological management of PH have introduced targeted therapies that modulate pathways involved in pulmonary vascular remodeling and vasodilation. One such therapeutic agent, riociguat, a soluble guanylate cyclase (s-GC) stimulator, has garnered attention for its efficacy in improving hemodynamic parameters and exercise capacity in PH patients.^[8]^ The s-GC serves as a pivotal mediator in the cardiovascular system, catalyzing the conversion of nitric oxide (NO) into cyclic guanosine monophosphate (cGMP), eliciting vasodilation and other beneficial effects.^[9]^ Riociguat has a dual mode of action, directly stimulating s-GC independently of NO, and increasing the sensitivity of s-GC to NO.^[10]^ Moreover, riociguat increases the level of cGMP, resulting in vaso-relaxation and antiproliferative and antifibrotic effects, as shown in experimental models of pulmonary hypertension.^[11]^ Additionally, riociguat has shown superiority in patients who do not achieve treatment goals with PDE5 inhibitors, offering a safer and more effective alternative in those cases.^[12]^ Its ability to improve right ventricular function and reduce pulmonary vascular resistance, critical factors in long-term outcomes, further underscores its advantage over existing therapies, particularly in cases of inoperable or residual CTEPH.^[13]^

Despite the growing use of riociguat for PH, particularly in PAH and CTEPH, a significant gap in the literature persists regarding the impact of varying dosages on both efficacy and safety. Previous studies, though valuable, have been limited by small sample sizes, heterogeneous populations, and inconsistent focus on dose-response relationships. This has led to inconclusive findings on the optimal dosing strategy for maximizing therapeutic benefits while minimizing adverse effects such as hypotension. The lack of a comprehensive meta-analysis on riociguat’s dose variability exacerbates these uncertainties, leaving clinicians with limited guidance on how to optimize treatment. By synthesizing data from multiple studies, this meta-analysis aims to resolve these inconsistencies, providing a robust evaluation of riociguat’s efficacy and safety across different doses. Ultimately, this work seeks to offer a practical solution to the real-world challenge of dose optimization, equipping clinicians with evidence-based insights to improve patient outcomes in PH management.

## 2. Materials and methods

This systematic review and meta-analysis adhered to the guidelines established by the preferred reporting items for systematic reviews and meta-analyses (PRISMA).^[14]^

### 2.1. Search and data sources

We conducted a thorough search across 4 prominent online databases: PubMed, Cochrane Central, Google Scholar, and Embase. Our aim was to include all relevant Randomized Controlled Trials (RCTs) published until May 5th, 2024. To identify relevant articles, we employed a comprehensive search strategy using keywords and Medical Subject Heading (MeSH) terms for “Riociguat” AND “Pulmonary Hypertension” the detail search string is available in Table S1, Supplemental Digital Content, https://links.lww.com/MD/Q723. The conference proceedings from the last 5 annual meetings of the American Thoracic Society (ATS) were hand-searched.

**Figure 1.**
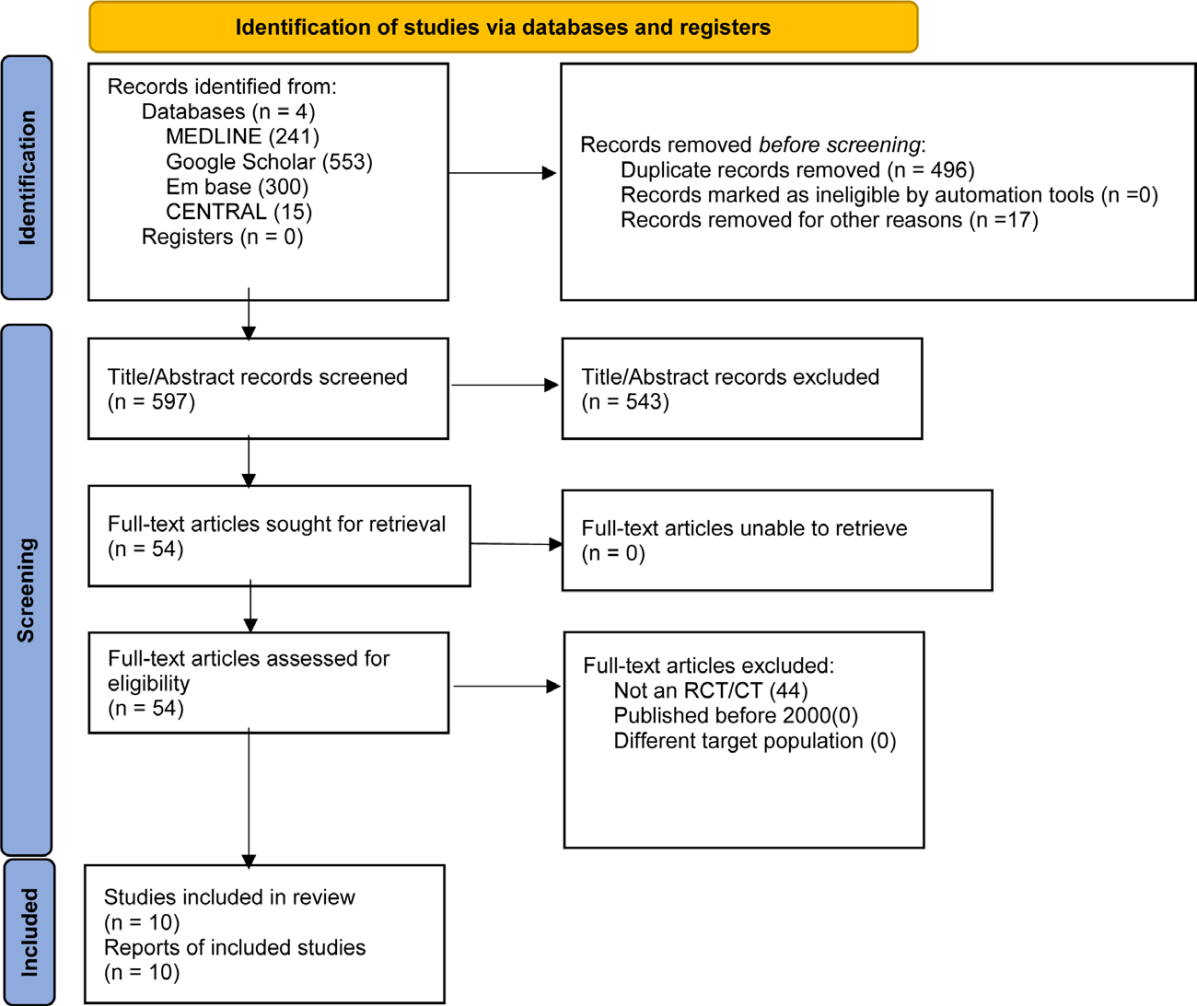
PRISMA flow diagram explaining search results and study selection process. CI = confidence interval, PRISMA = preferred reporting items for systematic reviews and meta-analyses, RCT = randomized controlled trial.

Bibliographies of the included original articles were also searched. We also performed forward citation searching using the Web of Science to identify further eligible articles. The PRISMA flowchart, illustrating the search results, can be found in Figure 1.

### 2.2. Eligibility criteria

To be included, studies had to meet predefined eligibility criteria. We focused exclusively on double-arm randomized control trials (RCTs) involving patients diagnosed with any form of pulmonary hypertension. Selected studies compared riociguat against placebo, with riociguat dosages ranging from 0.5 to 2.5 mg. Primary outcomes centered on reducing symptoms of pulmonary arterial hypertension. Excluded studies were those not in English, as well as review articles, case reports, case series, cross-sectional studies, editorials, commentaries, and animal studies.

### 2.3. Study selection and data extraction

Following the search, we used EndNote Reference Library software to identify and remove duplicate articles. Two authors independently screened titles and abstracts, followed by a full-text review to assess eligibility. Any discrepancies were resolved through consultation with a third author.

Two authors undertook data extraction, and the data was discussed with a third researcher in case of disagreement. Data was meticulously extracted and organized in an online Microsoft Excel Spreadsheet, including baseline characteristics such as the first author’s name, publication year, study design, gender, BMI, WHO functional class, 6-minute walk distance (6-MWD), and additional treatment for pulmonary arterial hypertension. Detailed study baseline characteristics are available in Table 1

**Table 1:** Study characteristic table demonstrating characteriscts of the included randomized controlled trials in meta-analysis.

First author and yr	Study type	Experimental/control	Etiology	Doses	Total (N)	Female, n (%)	Male, n(%)	Age, yr	BMI (kg/m^2^)	WHO functional class				6-MWD (m)	Additional treatment for pulmonary arterial hypertension	
										I	II	III	IV		Endothelin receptor antagonists	Prosta-noids
Ghofrani 2013^[8]^	RCT	Riociguat/placebo	PAH	Riociguat 2.5 mg dose 3 times daily	254	203 (80)	N/A	51 ± 17	26 ± 5	5 (2)	108 (43)	140 (55)	1 (<1)	361 ± 61	113 (44)	18 (7)
				Riociguat 1.5 mg dose 3 times a day	63	49 (78)	N/A	49 ± 16	27 ± 5	5 (8)	19 (30)	39 (62)	0	363 ± 67	27 (43)	4 (6)
			PAH-CHD	Placebo	126	98 (78)	N/A	51 ± 17	26 ± 5	4 (3)	60 (48)	58 (46)	3 (2)	368 ± 75	54 (43)	6 (5)
Rosenkranz 2015^[15]^	RCT	Riociguat/placebo		Riociguat 2.5 mg 3 times daily	15	13 (87)	N/A	35 ± 14	21 ± 2	N/A	67	33	N/A	369 ± 78	5 (33)	2 (13)
			Treatment naive	Riociguat 1.5 mg 3 times daily	8	6 (75)	N/A	41 ± 15	24 ± 5	N/A	50	50	N/A	391 ± 59	4 (50)	0
			Pretreated patients	Placebo	12	10 (83)	N/A	40 ± 16	24 ± 3	N/A	58	42	N/A	360 ± 59	3 (25)	1 (8)
Galiè 2017^[16]^	RCT	Riociguat/placebo	Inoperable CTEPH	Riociguat 2.5 mg 3 times daily (Tx naive patients)	123	94 (76)	N/A	48 (17)	25 ± 5	3 (2)	65 (53)	55 (45)	0 (0)	370 (66)	N/A	N/A
			Persistent or recurrent PH following PEA	Placebo (Tx naive patients)	66	52 (79)	N/A	48 ± 18	26 ± 6	4 (6)	35 (53)	25 (38)	2 (3)	360 (80)	N/A	N/A
			Idiopathic interstitial pneumonia associated PAH	Rocioguat 2.5 mg 3 times daily (pretreated patients overall)	131	109 (83)	N/A	54 ± 15	27 ± 6	2 (2)	43 (33)	85 (65)	1 (1)	353 (69)	N/A	N/A
			PAH caused by LV systolic dysfunction	Placebo(pretreated patients overall)	60	46 (77)	N/A	53 ± 15	27 ± 6	0 (0)	25 (42)	33 (56)	1 (2)	379 (68)	N/A	N/A
Kim 2017^[17]^	RCT	Riociguat/placebo		Rociguat 0.5–2.5 3 times daily (inoperable CTEPH)	121	86 (71)	N/A	59 ± 14	27 ± 5	2 (2)	38 (31)	75 (62)	6 (5)	335 (83)	N/A	N/A
			PAH caused by LV diastolic dysfunction	Placebo (inoperable CTEPH)	68	45 (66)	N/A	60 ± 12	28 ± 5	0 (0)	18 (26)	49 (72)	1 (1)	351 (75)	N/A	N/A
			CTEPH	Rociguat 0.5–2.5 3 times daily (persistent/recurrent PH after PEA)	52	32 (62)	N/A	60 ± 14	28 ± 7	1 (2)	17 (33)	36 (62)	2 (4)	360 (78)	N/A	N/A
			Sarcoidosis associated PAH	Placebo(Persistent/recurrent PH after PEA)	20	9 (45)	N/A	57 ± 15	28 ± 6	0 (0)	7 (35)	11 (55)	1 (5)	374 (72)	N/A	N/A
Nathan 2019^[18]^	RCT	Riociguat/placebo	PH in heart failure with preserved ejection fraction	Riociguat 2.5 mg 3 times daily	73	23 (32)	50 (68)	68 ± 8	30 ± 5	N/A	16 (22)	50 (68)	7 (10)	307 ± 80	N/A	N/A
				Placebo	74	29 (39)	45 (61)	69 ± 8	28 ± 6	N/A	22 (30)	45 (61)	7 (9)	324 ± 66	N/A	N/A
Bonderman 2013^[19]^	RCT	Riociguat/placebo		Riociguat 2 mg 3 times daily	67	N/A	55 (82)	59.3 (26.0–76.0)	28.9 (0.6)	35 (52)	31 (46)	1 (1)	N/A	411 ± 15 (n = 54)	N/A	N/A
				Riociguat 1 mg 3 times daily	33	N/A	30 (91)	N/A	28.2 (0.8)	20 (61)	13 (39)	0	N/A	420 ± 19 (n = 28)	N/A	N/A
				Placebo	69	N/A	61 (88)	59.3 (26.0–76.0)	28.7 (0.7)	42 (61)	23 (33)	4 (6)	N/A	414 ± 16 (n = 55)	N/A	N/A
Bonderman 2014^[20]^	RCT	Riociguat/placebo		Riociguat 2 mg 1 time daily	10		50	72.8 (59.0–83.0)	29.3 (23.5–33.4)	N/A	N/A	N/A	N/A	N/A	N/A	N/A
				Placebo	11		45	75.1 (65.0–86.0)	30.2 (21.8–36.0)	N/A	N/A	N/A	N/A	N/A	N/A	N/A
Ghofrani 2013^[8]^	RCT	Riociguat/placebo		Riociguat 0.5 mg to 2.5 mg 3 times daily	173	118 (68)		59 ± 14	27 ± 6	3	55	107	8	342 ± 82	N/A	N/A
				Placebo	28	54 (61)		59 ± 13	28 ± 5	0	25	60	2	356 ± 75	N/A	N/A
Baughman 2022^[21]^	RCT	Riociguat/placebo		Riociguat 0.5 to 2.5 mg 3 times daily		6	2	52 ± 7.0		N/A	N/A	N/A	N/A	271 ± 95.8	N/A	N/A
				Placebo		8	0	64 ± 6.3		N/A	N/A	N/A	N/A	332 ± 66.7	N/A	N/A
Dachs 2022^[22]^	RCT	Riociguat/placebo		Riociguat 1.5 mg 3 times daily	58	46 (79.3)	12 (20.7)	70.6 ± 8.0	32.1 ± 6.4	N/A	28 (56.0)	22 (44.0)		308.83 ± 109.41	N/A	N/A
				placebo	56	37 (66.1)	19 (33.9)	72.1 ± 8.5	30.3 ± 6.4	N/A	26 (48.1)	28 (51.9)		342.57 ± 116.14	N/A	N/A

### 2.4. Outcome measures

Primary outcomes for analysis included various parameters, 6-MWD, mean pulmonary arterial hypertension (mPAP), pulmonary vascular resistance (PVR), right atrial pressure (RAP), mean arterial pressure (MAP), cardiac index (CI), N-terminal pro-B-type Natriuretic peptide (NT-proBNP), systemic vascular resistance (SVR) and pulmonary artery wedge pressure (PAWP). Secondary outcomes covered adverse events and clinical worsening.

### 2.5. Effect measures

For categorical outcomes, the number of events for that outcome and the total number of patients were extracted, while for continuous outcomes, the sample size, means and standard divisions were extracted.

### 2.6. Quality assessment

**Figure 2.**
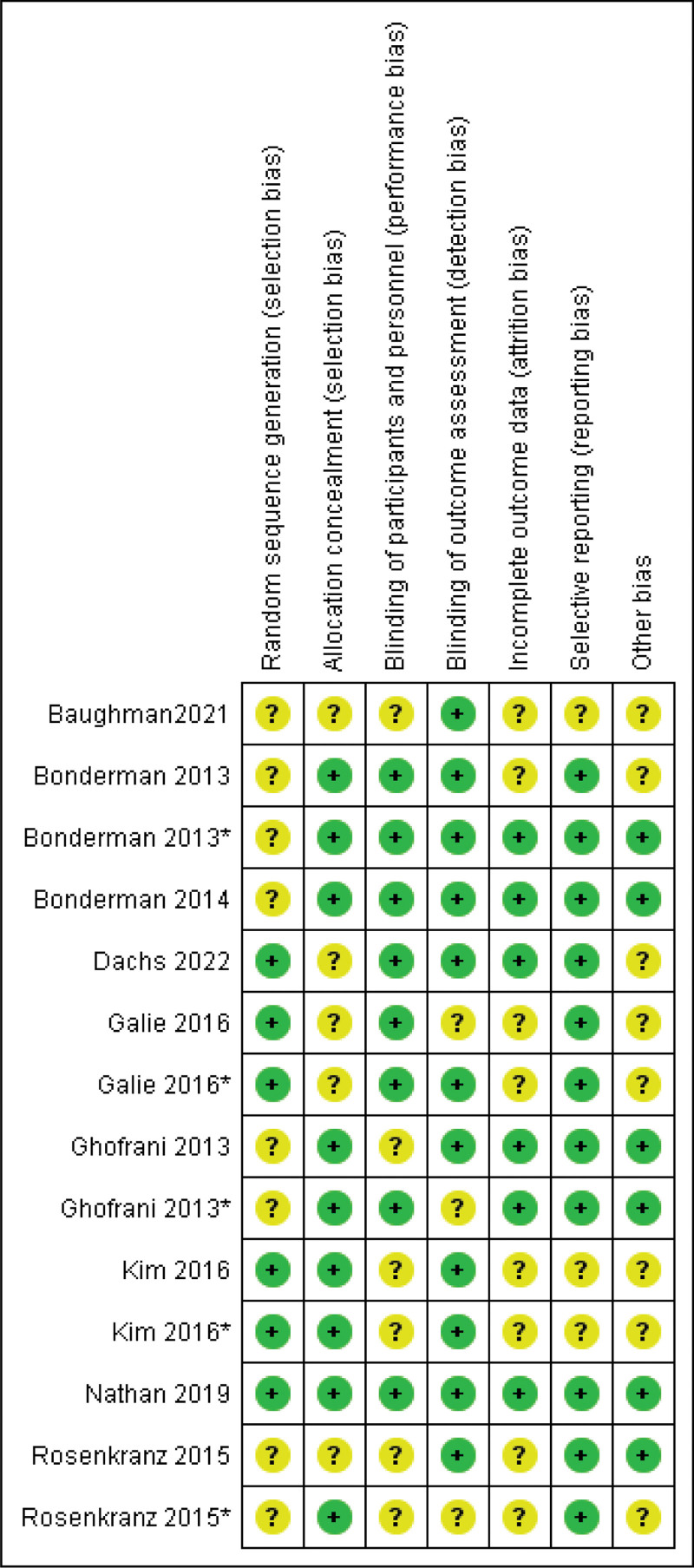
Summary of risk of assessment of the included randomized controlled trials in meta-analysis.

The quality of the included studies was evaluated using the Cochrane risk of bias (RoB) tool. Cochrane is a tool for measuring risk assessment that assigns a high-risk, low-risk, or unclear-risk assessment on key points. Two researchers independently assessed the RCTs for allocation sequence generation, participant randomization, blinding of patients and outcome data, selective outcome reporting, and missing data.^[23]^ The summary of the risk of bias assessment is illustrated in Figure 2, with the risk of bias graph available in Figure S1, Supplemental Digital Content, https://links.lww.com/MD/Q723.

### 2.7. Statistical analysis

We conducted the statistical analysis using Review Manager (RevMan, version 5.4; The Cochrane Collaboration, Copenhagen, Denmark) calculating risk ratios (RR) and mean differences (MD) for dichotomous and continuous outcomes respectively with associated 95% confidence intervals (CIs). A random-effects model Mantel–Haenszel method was employed to address heterogeneity among studies.^[24]^ The pooled results were represented graphically as forest plots Figure S2, Supplemental Digital Content, https://links.lww.com/MD/Q723. Significance was set at *P* ≤ .05, *χ*^2^ test and Higgins *I*^2^ statistics were used to report heterogeneity across the studies. *I*^2^ values were interpreted according to the Cochrane Handbook of Systematic Review of Interventions, section 10.10.^[25]^ Due to the high heterogeneity observed in all outcomes, a meta-regression analysis was also conducted to investigate the impact of covariates, specifically age and BMI, on the pooled effect sizes. Figure S3, Supplemental Digital Content, https://links.lww.com/MD/Q723.

### 2.8. Publication bias

The funnel plot was used to detect publication bias because the number of literature included was equal to 10. All estimates were reported with a 95% confidence interval (CI) using the random-effects model. Funnel plots for primary outcomes can be found in the Figure S3, Supplemental Digital Content, https://links.lww.com/MD/Q723.

### 2.9. Ethical consideration

This study is a systematic review and meta-analysis of previously published data. Since no new patient data were collected or directly involved, approval from an institutional review board/ethics committee and informed consent were not required.

## 3. Results

### 3.1. Literature search and study selection

The thorough screening through the 4 included databases including: PubMed, Cochrane Central, Google Scholar, and Embase, yielded a total of 1109 articles. After the exclusion of duplicate records, there were 597 studies subject to title-and-abstract level screening by independent investigators. Subsequently, 54 studies were subject to full-text review. Ultimately, there were 10 studies declared as eligible for inclusion within this qualitative and quantitative synthesis.^[8,15–22,26]^ The study selection process is illustrated in the PRISMA flowchart in Figure 1.

### 3.2. Study baseline characteristics

In this systematic review and meta-analysis, data from multiple studies evaluating riociguat therapy for pulmonary hypertension (PH) were synthesized, focusing on patient characteristics across different etiologies of PH. The analysis included 10 studies, featuring a total of 1189 patients treated with riociguat at varying doses (ranging from 0.5 to 2.5 mg, administered 1 to 3 times daily) and 658 in the placebo arm. The mean age of the patients 50.6 spanned from 36 to 70 years and 58.9 spanned from 40 to 75.1 years respectively for the riociguat and placebo arms. The mean body mass index (BMI) was 26.8, ranging from 21 to 32.1 kg/m^2^ for the Riociguat group, it was 27.7, ranging from 24 to 30.3 for placebo group. Across all trials, the baseline 6-minute walk distance (6-MWD) varied between 271 and 401 meters in the riociguat arm, reflecting differences in baseline functional capacity depending on the PH subtype. The study population baseline characteristics are available in Table 1.

### 3.3. Risk of bias assessment

Most studies demonstrated a low-risk of selection bias, with well-reported random sequence generation and allocation concealment. Blinding, a critical factor in reducing performance and detection biases, was well-maintained across studies, with nearly all trials ensuring that participants, personnel, and outcome assessors were blinded. This uniformity in blinding practices supports the internal validity of the studies, reinforcing the reliability of riociguat’s reported effects. Attrition bias was minimal, as most studies effectively managed incomplete outcome data with clear documentation, which strengthens the overall data integrity and limits the likelihood of bias from participant dropouts. Selective reporting was consistently low, with outcomes prespecified and comprehensively reported, thereby reducing the risk of reporting bias and enhancing transparency in data presentation. A minor concern regarding other potential biases was noted in specific studies, although these were isolated and did not materially affect the pooled findings. The summary of the risk of bias assessment is illustrated in Figure 2, with the risk of bias graph available in Figure S1, Supplemental Digital Content, https://links.lww.com/MD/Q723.

### 3.4. Six-minute walk distance (6MWD)

The forest plot presents the results of 9 trials,^[8,26,15,18–22]^ evaluating the impact of different doses of riociguat, compared to placebo, on the 6-minute walk distance (6MWD) in patients with pulmonary hypertension. The analysis includes fixed doses as well as dose escalation. Overall, the aggregated data from all dosages of riociguat compared to placebo yielded a pooled mean difference in 6MWD of 27.23 meters (95% CI: 8.28 to 46.18; *P* = .005), signifying a significant improvement favoring riociguat. Riociguat’s efficacy in improving 6MWD appears to be dose-dependent. The 2.5 mg 3 times daily dose shows the most consistent and significant improvement. Lower doses (1 and 2 mg 3 times daily) either show no improvement or a potential decrease in 6MWD. However, the dose escalation regimen (0.5–2.5 mg 3 times daily) shows significant improvement with riociguat, although with some heterogeneity.

Nonetheless, the overall heterogeneity was notably high (*I*^2^ = 89%, *P* < .00001), indicating variability among the included studies, necessitating further investigation. Given the high heterogeneity observed, a meta-regression analysis was conducted to explore the influence of age and BMI on 6MWD associated with pulmonary hypertension (PH) in patients receiving riociguat therapy. BMI was significantly negatively associated with 6MWD (Coefficient: −8.782, 95% CI: −15.39 to −2.174, *P* = .009), indicating that higher BMI is associated with reduced exercise capacity in PH patients on riociguat therapy. However, age did not show a statistically significant effect on 6MWD (Coefficient: −1.161, 95% CI: −2.856 to 0.535, *P* = .18).

### 3.5. Mean pulmonary artery pressure (mPAP)

The forest plot presents the results of 12^[8,15–17,26,19,20,22]^ studies evaluating the impact of different doses of riociguat, compared to placebo, on the mean pulmonary artery pressure (mPAP). The overall analysis of all included dosages of riociguat versus placebo revealed a pooled mean difference in mPAP of −2.61 mm Hg (95% CI: −4.60 to −0.63; *P* = .01), indicating a significant reduction in mPAP with riociguat. Riociguat’s efficacy in reducing mPAP appears to be dose-dependent. The 2 and 2.5 mg 3 times daily fixed doses, and the dose escalation regimen show the most significant improvements. However, lower fixed doses (1 mg 3 times daily and 2 mg once daily) show either no improvement or a potential detrimental effect (increase in mPAP). Strikingly, The dose escalation regimen (0.5–2.5 mg 3 times daily) shows the most significant reduction in mPAP with no heterogeneity, indicating a strong and consistent beneficial effect.

Nevertheless, the overall heterogeneity was notably high (*I*^2^ = 91%, *P* < .00001), suggesting considerable variability among the studies. Given the high heterogeneity observed, a meta-regression analysis was conducted to explore the influence of age and BMI on mPAP associated with pulmonary hypertension (PH) in patients receiving riociguat therapy. Neither age (coefficient: 0.13, 95% CI: −0.107 to 0.368, *P* = .282) nor BMI (coefficient: 0.731, 95% CI: −0.338 to 1.8, *P* = .18) demonstrated a significant association with mPAP.

### 3.6. Pulmonary vascular resistance

The forest plot presents the results of 13^[8,15–17,26,19–22]^ studies evaluating the impact of different doses of riociguat, compared to placebo, on the pulmonary vascular resistance. The overall pooled analysis demonstrated a significant reduction in PVR with riociguat treatment. The weighted mean difference (WMD) for PVR across all studies was −90.27 dyn·s·cm^−5^ (95% CI: −119.30 to −61.23; *P* < .00001), indicating a robust reduction favoring riociguat over placebo. Riociguat’s efficacy in reducing PVR appears to be dose-dependent. The 2 and 2.5 mg 3 times daily fixed doses show the most significant improvements. Lower fixed doses (1 mg 3 times daily and 2 mg once daily) show no significant improvement in PVR. The dose escalation regimen (0.5–2.5 mg 3 times daily) shows a nonsignificant reduction in PVR with high heterogeneity, indicating variability in individual responses.

Significant heterogeneity was present (*I*^2^ = 93%), reflecting variability among the included studies. Given the high heterogeneity observed, a meta-regression analysis was conducted to explore the influence of age and BMI on PVR associated with pulmonary hypertension (PH) in patients receiving riociguat therapy. No significant associations were observed for age (coefficient: 7.121, 95% CI: −1.297 to 15.539, *P* = .097) or BMI (coefficient: 11.515, 95% CI: −24.577 to 47.607, *P* = .532) with PVR, although age trended toward significance.

### 3.7. Right atrial pressure (RAP)

The forest plot presents the results of 13^[8,15–17,26,19–22]^ studies evaluating the impact of different doses of riociguat, compared to placebo, on the mean arterial pressure. Riociguat at a dose of 2.5 mg 3 times daily appears to have a significant and consistent effect in reducing RAP in patients with pulmonary hypertension. Other dosing regimens either showed inconsistent effects or nonsignificant changes in RAP.

We observed high (*I*^2^ = 72%) heterogeneity across all doses and studies. Upon conducting a meta-regression analysis age did not show a significant association with RAP (coefficient: 0.122, 95% CI: −0.004 to 0.249, *P* = .058), while BMI trended toward significance (coefficient: 0.466, 95% CI: 0.069 to 0.864, *P* = .022), indicating a potential positive association between higher BMI and increased RAP.

### 3.8. Mean arterial pressure (MAP)

The forest plot presents the results of 8 studies^[8,26,16,17,19,20]^ evaluating the impact of different doses of riociguat, compared to placebo, on the mean arterial pressure. The overall mean difference of −3.77 mm Hg (95% CI: −8.72 to 1.17, *P* = .13), suggests no significant overall effect of riociguat on MAP when compared to placebo. However, the 2.5 mg dose taken 3 times daily showed the most significant reduction in MAP, suggesting it may be the most effective regimen among those studied. While, the 2 mg once daily dose also showed a significant reduction, although less pronounced than the 2.5 mg 3 times daily dose. Moreover, dose escalation regimens and lower doses (1 mg 3 times daily) did not show a consistent or significant effect.

The heterogeneity across all studies was notably high (*I*^2^ = 96%), indicating significant variability among study outcomes. Given the high heterogeneity observed, a meta-regression analysis was conducted to explore the influence of age and BMI on MAP associated with pulmonary hypertension (PH) in patients receiving riociguat therapy. Both age (coefficient: 0.059, 95% CI: −0.04 to 0.157, *P* = .244) and BMI (coefficient: 0.509, 95% CI: −0.133 to 1.152, *P* = .12) were not significantly associated with MAP.

### 3.9. Cardiac index

The forest plot presents the results of 11^[15–22]^ studies evaluating the impact of different doses of riociguat, compared to placebo, on the cardiac index. The overall effect size indicated a significant improvement in cardiac index with riociguat, showing a mean difference of 0.36 L/min/m^2^ (95% CI: 0.19 to 0.53, *P* < .0001). The analysis indicates a dose-dependent effect of riociguat on the CI. Higher doses (2 mg and above) and dose escalation show significant positive effects, while lower doses (1.5 mg and below) do not show significant improvements.

The heterogeneity across all studies was moderate (*I*^2^ = 74%). Given the high heterogeneity observed, a meta-regression analysis was conducted to explore the influence of age and BMI on CI associated with pulmonary hypertension (PH) in patients receiving riociguat therapy. The associations between CI and age (coefficient: −0.009, 95% CI: −0.027 to 0.009, *P* = .323) or BMI (coefficient: −0.042, 95% CI: −0.125 to 0.041, *P* = .318) were not statistically significant.

### 3.10. Cardiac output

The forest plot presents the results of 8 studies^[8,26,16,17,20,22]^ evaluating the impact of different doses of riociguat, compared to placebo, on the cardiac index. The aggregated data from all dosages of riociguat compared to placebo yielded a pooled mean difference in cardiac output of 0.85 L/min (95% CI: 0.70 to 1.00; *P* < .00001), signifying a significant improvement favoring riociguat. The 2.5 mg dose of riociguat taken 3 times daily showed the most significant increase in CO compared to placebo. Riociguat at a dose of 1.5 mg 3 times daily and 2 mg once daily did not show statistically significant differences in CO compared to placebo. Dose escalation regimens (0.5 to 2.5 mg 3 times daily) also showed a significant increase in CO compared to placebo.

Nonetheless, the overall heterogeneity was low to moderate (I^2^ = 31.7%, *P* = .26), indicating some variability among the included studies. Upon conducting a meta-regression analysis both age (coefficient: −0.024, 95% CI: −0.047 to −0.002, *P* = .032) and BMI (coefficient: −0.114, 95% CI: −0.216 to −0.013, *P* = .027) demonstrated significant negative associations with CO, indicating that increased age and BMI may contribute to reduced cardiac output.

### 3.11. N-terminal pro-type B natriuretic peptide (NT-proBNP)

The forest plot presents the results of 7 studies^[8,26,15,19,22]^ evaluating the impact of different doses of riociguat, compared to placebo, on the NT-proBNP. Overall, the aggregated data from all dosages of riociguat compared to placebo yielded a pooled mean difference in NT-proBNP levels of −256.77 pg/mL (95% CI: −491.99 to −21.55; *P* = .03), signifying a significant reduction favoring riociguat. The 1 and 2 mg dose of riociguat taken 3 times daily showed the most significant decrease in NT-proBNP levels compared to placebo. Other doses do not show statistically significant differences in NT-proBNP levels compared to placebo.

Nonetheless, the overall heterogeneity was moderate (*I*^2^ = 31.7%, *P* = .31), indicating variability among the included studies. Upon conducting a meta-regression analysis age (coefficient: 15.684, 95% CI: 5.359–26.009, *P* = .003) and BMI (coefficient: 64.896, 95% CI: 20.112–109.68, *P* = .005) were both significantly positively associated with NTproBNP levels, suggesting higher levels of cardiac stress biomarkers in older and higher-BMI patients.

### 3.12. Systemic vascular resistance

The forest plot presents the results of 7 studies^[16,17,19,22]^ evaluating the impact of different doses of riociguat, compared to placebo, on the NT-proBNP. the combined analysis revealed a significant reduction in SVR with riociguat, with an overall mean difference of −197.16 dyn·s·cm^−5^ (95% CI: −331.28 to −63.03, *P* = .004). The most significant reduction in SVR was seen with the dose escalation and 2.5 mg dose of riociguat, taken 3 times daily. Riociguat at a dose of 2 mg once daily and 1 mg 3 times daily did not show statistically significant differences in SVR compared to placebo.

However, significant heterogeneity was present across the studies (*P* < .00001; *I*^2^ = 93%). Given the high heterogeneity observed, a meta-regression analysis was conducted to explore the influence of age and BMI on SVR associated with pulmonary hypertension (PH) in patients receiving riociguat therapy. BMI was significantly associated with increased SVR (coefficient: 103.84, 95% CI: 8.812–198.869, *P* = .032), while age did not show a statistically significant effect (coefficient: 20.901, 95% CI: −8.616 to 50.417, *P* = .165).

### 3.13. Pulmonary artery wedge pressure

The forest plot presents the results of 8 studies^[8,26,17–19]^ evaluating the impact of different doses of riociguat, compared to placebo, on pulmonary artery wedge pressure. The aggregated data from all dosages of riociguat compared to placebo yielded a pooled mean difference in PAWP of 0.43 mm Hg (95% CI: −0.75 to 1.61, *P* = .47), indicating no significant overall effect of riociguat. A dosage of 2.5 mg 3 times daily and a dose escalation of 0.5–2.5 mg 3 times daily both showed no significant effect on PAWP without heterogeneity. Similarly, a 1.5 mg 3 times daily dosage showed no significant effect. In contrast, a 1 mg 3 times daily dosage significantly increased PAWP, while a 2 mg 3 times daily dosage significantly reduced PAWP.

Significant heterogeneity was present across the included studies (*I*^2^ = 89%, *P* < .00001), indicating variability among the included studies. Given the high heterogeneity observed, a meta-regression analysis was conducted to explore the influence of age and BMI on PAWP associated with pulmonary hypertension (PH) in patients receiving riociguat therapy. Neither age (coefficient: −0.038, 95% CI: −0.189 to 0.113, *P* = .621) nor BMI (coefficient: 0.025, 95% CI: −0.763–0.814, *P* = .95) were significantly associated with PAWP.

### 3.14. Any adverse events

The forest plot presents the results of 10 studies^[8,15,16,26,19,20,22]^ evaluating the impact of different doses of riociguat, compared to placebo, on the any treatment-emergent adverse events. A pooled risk ratio of 0.99 (95% CI: 0.91–1.08, *P* = .80), suggests no significant overall difference in the incidence of adverse events between riociguat and placebo. The 2.5 mg 3 times daily dosage showed no significant difference in the risk of adverse events compared to placebo, with no observed heterogeneity. Similarly, the 0.5 to 2.5 mg 3 times daily dosage indicated no significant difference in adverse event rates with no heterogeneity. The 1.5 mg 3 times daily dosage also showed no significant difference in adverse events with negligible heterogeneity. Conversely, the 1 mg 3 times daily dosage demonstrated a significant reduction in adverse events with no heterogeneity, while the 2 mg 3 times daily and 2 mg once daily dosages showed no significant difference in adverse event rates. Significant heterogeneity was observed across the studies (*I*^2^ = 67%).

### 3.15. Clinical worsening outcome

When combining the data across all 3 studies,^[8,26,15]^ the overall risk ratio was 0.40 (95% CI: 0.11–1.44, *P* = .16), indicating no significant overall difference in the risk of clinical worsening with riociguat compared to placebo. The heterogeneity across the studies was moderate (τ^2^ = 0.53, *χ*^2^ = 3.51, *df* = 2, *P* = .17; *I*^2^ = 43%), suggesting some variability in the study outcomes.

### 3.16. Publication bias

Funnel plots were made to evaluate the presence of publication bias. The funnel plots visually displayed the distribution of studies for each outcome, with the vertical axis representing the effect size and the horizontal axis representing the precision of the estimates. In general, the funnel plots exhibited an equal and symmetrical distribution of studies on both sides of the vertical axis, indicating no significant publication bias for most outcomes.

However, it is worth noting that only 1 outcome, the cardiac index, displayed an asymmetric distribution of studies, with an apparent lack of smaller studies reporting more extreme effect sizes. This observation suggests a potential publication bias in these specific outcomes.

## 4. Discussion

Our meta-analysis provides the first comprehensive evaluation of the effects of varying riociguat dosages on key hemodynamic and clinical outcomes in patients with pulmonary arterial hypertension (PAH) and chronic thromboembolic pulmonary hypertension (CTEPH). The findings confirm a clear dose-dependent response, with the 2.5 mg dose taken 3 times daily delivering the most consistent and significant improvements. This optimal dosage resulted in notable enhancements in 6-minute walk distance (6MWD), mean pulmonary artery pressure (mPAP), pulmonary vascular resistance (PVR), cardiac output (CO), and right atrial pressure (RAP). In contrast, lower doses of 1 and 2 mg had limited to no effects, emphasizing the critical importance of dose optimization. The long-term observational study by Yang et al supports these findings, showing sustained 6MWD improvements over 96 months with the 2.5 mg dose, which aligns with the dose-dependent response observed in our study. This highlights the importance of maintaining optimal riociguat dosing to achieve lasting therapeutic benefits.^[27]^

In terms of pulmonary hemodynamics, the pooled analysis showed significant reductions in mPAP with riociguat, consistent with findings from Marra et al, which highlighted that long-term riociguat treatment not only lowers mPAP but also reduces right heart size and enhances right ventricular function.^[28]^ Preclinical data from Lang et al further reinforce this, showing that higher doses of riociguat significantly decrease right ventricular systolic pressure (RVSP), a surrogate for mPAP.^[11]^ This consistency across clinical and experimental studies suggests that riociguat effectively targets the NO-sGC-cGMP pathway, resulting in improved hemodynamic and cardiac outcomes.

The results also demonstrated a significant reduction in PVR with riociguat, particularly at higher fixed doses (2 and 2.5 mg 3 times daily). These improvements align with previous studies, such as those by Jaïs et al, which demonstrate that riociguat not only reduces PVR but is also effective when compared to balloon pulmonary angioplasty.^[29]^ In contrast, lower doses and dose escalation regimens (0.5–2.5 mg 3 times daily) showed nonsignificant reductions, with high heterogeneity possibly stemming from differences in patient responses or disease severity. Compared to existing literature, the consistency of results at higher doses supports the notion that sustained PVR reduction requires optimized dosing, while variability at lower or escalating doses may arise from inconsistent therapeutic levels or patient-specific factors.

Regarding right atrial pressure (RAP), our findings indicate that the 2.5 mg dose consistently reduced RAP, while other dosing regimens yielded variable or nonsignificant effects. This is consistent with studies such as Marra et al, which showed that long-term riociguat treatment significantly reduced right atrial area, suggesting a decrease in RAP. Similarly, the study reported improvements in right heart function correlating with atrial pressures. The alignment between our study and the existing research is likely due to the long-term use of riociguat, which has been shown to reduce right heart size and enhance right ventricular function, as demonstrated in Marra et al.^[28]^

In terms of mean arterial pressure (MAP), our analysis did not show an overall significant effect of riociguat compared to placebo. However, the 2.5 mg dose did show significant reduction in MAP, suggesting that it may be the most effective regimen. This aligns with the findings from Ghofrani et al, which reported that higher riociguat doses, particularly 2.5 mg, significantly reduced MAP in patients with pulmonary hypertension.^[30]^ However, unlike the pharmacokinetic analysis by Saleh et al, which found riociguat’s hemodynamic effects to be consistent across doses, our study observed variability, especially with lower or escalating doses, suggesting that riociguat’s effectiveness on MAP may be more dose-dependent than previously reported.^[31]^

Our analysis also highlighted a significant improvement in cardiac index with riociguat, particularly at higher doses (2 mg and above), indicating a dose-dependent response. This finding is consistent with the results from Yang et al, where 95% of patients on the 2.5 mg dose showed significant increases in cardiac index. Similarly, other trials reported improvements in cardiac function with higher doses of riociguat.^[27]^ The lack of significant improvement at lower doses in our study may be due to differences in study design or shorter follow-up periods, as existing studies generally focused on long-term treatment, which likely allowed more time for riociguat to exert its full hemodynamic benefits. These results suggest that longer treatment durations and higher doses are critical to achieving sustained improvements in cardiac function.

In addition, our analysis showed a significant improvement in cardiac output (CO) with riociguat, especially with the 2.5 mg dose taken 3 times daily, while lower doses (1.5 and 2 mg once daily) did not show significant changes. This aligns with previous research, such as studies by Ahmadi et al and Murata et al, which reported increased right ventricular stroke volume and improvements in cardiac index and right ventricular function with riociguat treatment.^[32,33]^ The consistency of these findings suggests that riociguat enhances CO by improving right ventricular contractility, as observed across several studies. The differences in outcomes at lower doses likely reflect insufficient therapeutic levels needed to achieve the same hemodynamic benefits, further emphasizing the importance of optimizing dosing to consistently improve CO.

We also found a significant reduction in NT-proBNP levels with riociguat, particularly at 1 and 2 mg doses taken 3 times daily, while other doses did not show significant reductions. This contrasts with existing studies, such as Liu et al and Zhang et al, which used mixed dosing regimens and found no significant reduction in NT-proBNP.^[34,35]^ This variation may stem from our use of fixed doses in the analysis, while the use of mixed regimens in other studies could have diluted the effect on NT-proBNP. These findings suggest that specific, consistent dosing is more effective in achieving reductions in NT-proBNP levels.

Our analysis of systemic vascular resistance (SVR) also revealed a significant reduction with riociguat, particularly with the dose escalation regimen and the 2.5 mg dose taken 3 times daily. Lower doses did not show significant effects. This contrasts with the existing literature, such as the study by Bonderman et al, which found no significant change in SVR but noted improvements in stroke volume, systolic blood pressure, and right ventricular function. The focus on higher, consistent dosing in our study likely contributed to the observed reduction in SVR, supporting the notion that optimal dosing plays a key role in achieving vascular improvements.^[20]^

Additionally, our study showed no significant overall effect of riociguat on pulmonary arterial wedge pressure (PAWP) across various doses. Interestingly, the 1 mg 3 times daily dose increased PAWP, while the 2 mg dose reduced it. This contrasts with a study, where patients switching from phosphodiesterase-5 inhibitors to riociguat showed clinical improvements, suggesting riociguat may help manage PAWP in certain patients.^[36]^ The differing results may be due to the focus on specific patients not responding to previous therapies, indicating that the effects of riociguat on PAWP may depend on patient selection and dosing strategies.

Regarding safety, our analysis showed no significant difference in the incidence of adverse events between riociguat and placebo, which aligns with existing literature supporting riociguat’s favorable safety profile. Previous studies, such as those by Makowski et al, have demonstrated that riociguat is generally well-tolerated, with most patients able to tolerate maximal doses without serious adverse events.^[37]^ The consistency of our findings with prior research indicates that riociguat maintains a stable safety profile across different patient groups and dosing regimens, making it a reliable option for long-term treatment.

Riociguat plays a vital role in treating pulmonary arterial hypertension (PAH) and chronic thromboembolic pulmonary hypertension (CTEPH), significantly improving patients’ quality of life and long-term outcomes. Its efficacy in enhancing 6-minute walk distance (6MWD), reducing mean pulmonary artery pressure (mPAP), and improving right heart function underscores its potential to alleviate the hemodynamic burden of these conditions. Optimized dosing, particularly at 2.5 mg 3 times daily, has proven effective in studies demonstrating its benefits.^[38]^ Long-term usage has shown sustained improvements in functional capacity and exercise tolerance through its action on the NO-sGC-cGMP pathway, enhancing vasodilation and cardiac output.^[39]^ Riociguat’s favorable safety profile and dose flexibility make it a reliable long-term treatment option for managing PAH and CTEPH, helping to prolong survival and improve daily functioning.^[40,41]^

This meta-analysis also has several strengths. We adhered strictly to PRISMA guidelines, exclusively included RCTs, and conducted an exhaustive search across multiple databases, thereby minimizing bias and ensuring comprehensive coverage of relevant studies. By analyzing key clinical outcomes such as the 6-minute walk distance (6MWD), mean pulmonary artery pressure (mPAP), pulmonary vascular resistance (PVR), and cardiac function, we provide a comprehensive evaluation of riociguat’s effects. Our dose-specific analysis underscores the importance of optimizing doses, offering valuable insights for clinical decision-making. Additionally, the use of a random-effects model addresses heterogeneity, thereby improving the generalizability of our findings.

Despite the rigorous methodology used in this systematic review and meta-analysis, several limitations must be noted. The exclusion of non-English studies may have introduced language bias, potentially missing relevant research. While we aimed to include all relevant randomized controlled trials (RCTs), publication bias remains a concern, particularly regarding unpublished negative results. High heterogeneity in certain outcomes, such as dose-dependent effects, suggests that differences in study design, populations, and treatment durations may have influenced the findings. Additionally, reliance on reported data and variations in outcome definitions or measurement techniques could impact result comparability. Lastly, small sample sizes for some dosing regimens may limit the generalizability of specific subgroup findings.

Optimizing the 2.5 mg dose of riociguat, taken 3 times daily, is critical for achieving consistent improvements in hemodynamic outcomes like mPAP and PVR in patients with pulmonary arterial hypertension (PAH) and chronic thromboembolic pulmonary hypertension (CTEPH). Dosing should be personalized to meet individual patient needs to maximize both efficacy and safety. Research by Ghofrani et al emphasizes the dose-dependent enhancements in cardiac function.^[8]^ Future studies should focus on combination therapies, long-term safety, and utilizing biomarkers such as NT-proBNP for tailored treatment approaches

## 5. Conclusion

In conclusion, our meta-analysis confirms that riociguat is an effective treatment for improving key hemodynamic and functional outcomes in patients with PAH and CTEPH, particularly when using the optimal dose of 2.5 mg 3 times daily. The significant improvements in 6MWD, mPAP, PVR, and cardiac function highlight its therapeutic value. While lower doses showed limited benefits, the favorable safety profile of riociguat across various studies reinforces its suitability for long-term use. These findings support the use of optimized riociguat dosing to achieve better patient outcomes in PAH and CTEPH management.

## Acknowledgments

The authors would like to thank all researchers whose studies were included in this meta-analysis. No individuals are listed by name in the Acknowledgments section; therefore, no permission was required.

## Author contributions

**Conceptualization:** Muhammad Aqib Faizan, Tooba Rehman, Hurais Malik, Muhammad Hudaib, Zainab Humayun.

**Formal analysis:** Muhammad Aqib Faizan, Hurais Malik, Muhammad Hudaib.

**Methodology:** Muhammad Aqib Faizan, Tooba Rehman, Muhammad Abdullah Tahir.

**Supervision:** Zainab Humayun.

**Validation:** Zainab Humayun.

**Visualization:** Zainab Humayun.

**Writing – original draft:** Muhammad Aqib Faizan, Tooba Rehman, Muhammad Abdullah Tahir, Gunvi Ohri, Saher Bano, Laveeza Fatima.

**Writing – review & editing:** Hurais Malik, Muhammad Hudaib, Syed Hassan Ahmed, Ayat ul Karam, Zuhair Ahmed, Abdul Rehman, Samra Rabbani, Mrunalini Dandamudi, Mohammed Mahmmoud Fadelallah Eljack.

## Supplementary Material
