# Supplementary material for: Optimizing riociguat therapy for pulmonary hypertension: A systematic review and meta-analysis of dose variability, safety, and efficacy
Source: Medicine (Baltimore). 2026 Apr 24;105(17):e46007. doi: 10.1097/MD.0000000000046007 (PMC13124407; doi:10.1097/MD.0000000000046007)
Supplement: Supplementary file 1 [file medi-105-e46007-s001.pdf]

Figure S1, Supplementary Digital Material: Risk of Bias Graph

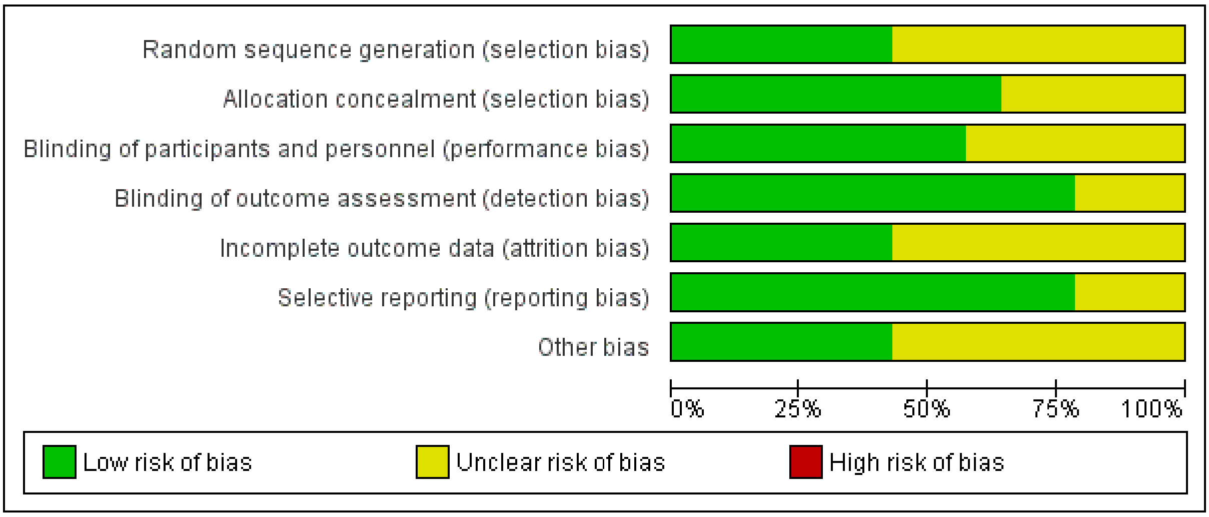

Supplementary Digital Material Figures S2: Forest Plot

Figures S2 (A): Six-minute Walk distance (6MWD)

Study or Subgroup	Riociguat Mean	Riociguat SD	Riociguat Total	Placebo Mean	Placebo SD	Placebo Total	Weight	Mean Difference IV, Random, 95% CI
**1.1.1 Riociguat 1.5mg three times daily**								
Dachs 2022	21.26	109.46	37	10.3	55.01	40	9.6%	10.96 [-28.21, 50.13]
Rosenkranz 2015*	43	54	8	0	42	12	8.6%	43.00 [-1.33, 87.33]
**Subtotal (95% CI)**			**45**			**52**	**18.3%**	**25.23 [-5.98, 56.44]**
Heterogeneity: Tau² = 57.79; Chi² = 1.13, df = 1 (P = 0.29); I² = 11%								
Test for overall effect: Z = 1.58 (P = 0.11)								
**1.1.2 Riociguat 2.5 mg three times daily**								
Ghofrani 2013	30	66	254	-6	86	126	14.5%	36.00 [18.93, 53.07]
Nathan 2019	38	133.6	31	-16	118.5	34	6.0%	54.00 [-7.63, 115.63]
Rosenkranz 2015	39	60	15	0	42	12	9.8%	39.00 [0.44, 77.56]
**Subtotal (95% CI)**			**300**			**172**	**30.2%**	**37.55 [22.42, 52.68]**
Heterogeneity: Tau² = 0.00; Chi² = 0.31, df = 2 (P = 0.86); I² = 0%								
Test for overall effect: Z = 4.86 (P < 0.00001)								
**1.1.3 Riociguat 1mg three times daily**								
Bonderman 2013	19	25.22	28	31	21.86	55	15.5%	-12.00 [-22.98, -1.02]
**Subtotal (95% CI)**			**28**			**55**	**15.5%**	**-12.00 [-22.98, -1.02]**
Heterogeneity: Not applicable								
Test for overall effect: Z = 2.14 (P = 0.03)								
**1.1.4 Riociguat 2mg three times daily**								
Bonderman 2013*	30.2	22.5	54	31	21.86	55	15.9%	-0.80 [-9.13, 7.53]
**Subtotal (95% CI)**			**54**			**55**	**15.9%**	**-0.80 [-9.13, 7.53]**
Heterogeneity: Not applicable								
Test for overall effect: Z = 0.19 (P = 0.85)								
**1.1.7 Riociguat 0.5 to 2.5mg three times daily**								
Baughman2021	39	34	7	-55	75.5	8	6.5%	94.00 [35.93, 152.07]
Ghofrani 2013*	39	79	173	-6	84	88	13.6%	45.00 [23.87, 66.13]
**Subtotal (95% CI)**			**180**			**96**	**20.1%**	**61.73 [16.19, 107.27]**
Heterogeneity: Tau² = 703.53; Chi² = 2.42, df = 1 (P = 0.12); I² = 59%								
Test for overall effect: Z = 2.66 (P = 0.008)								
**Total (95% CI)**			**607**			**430**	**100.0%**	**27.23 [8.28, 46.18]**
Heterogeneity: Tau² = 570.51; Chi² = 54.45, df = 8 (P < 0.00001); I² = 85%								
Test for overall effect: Z = 2.82 (P = 0.005)								
Test for subgroup differences: Chi² = 36.26, df = 4 (P < 0.00001), I² = 89.0%								

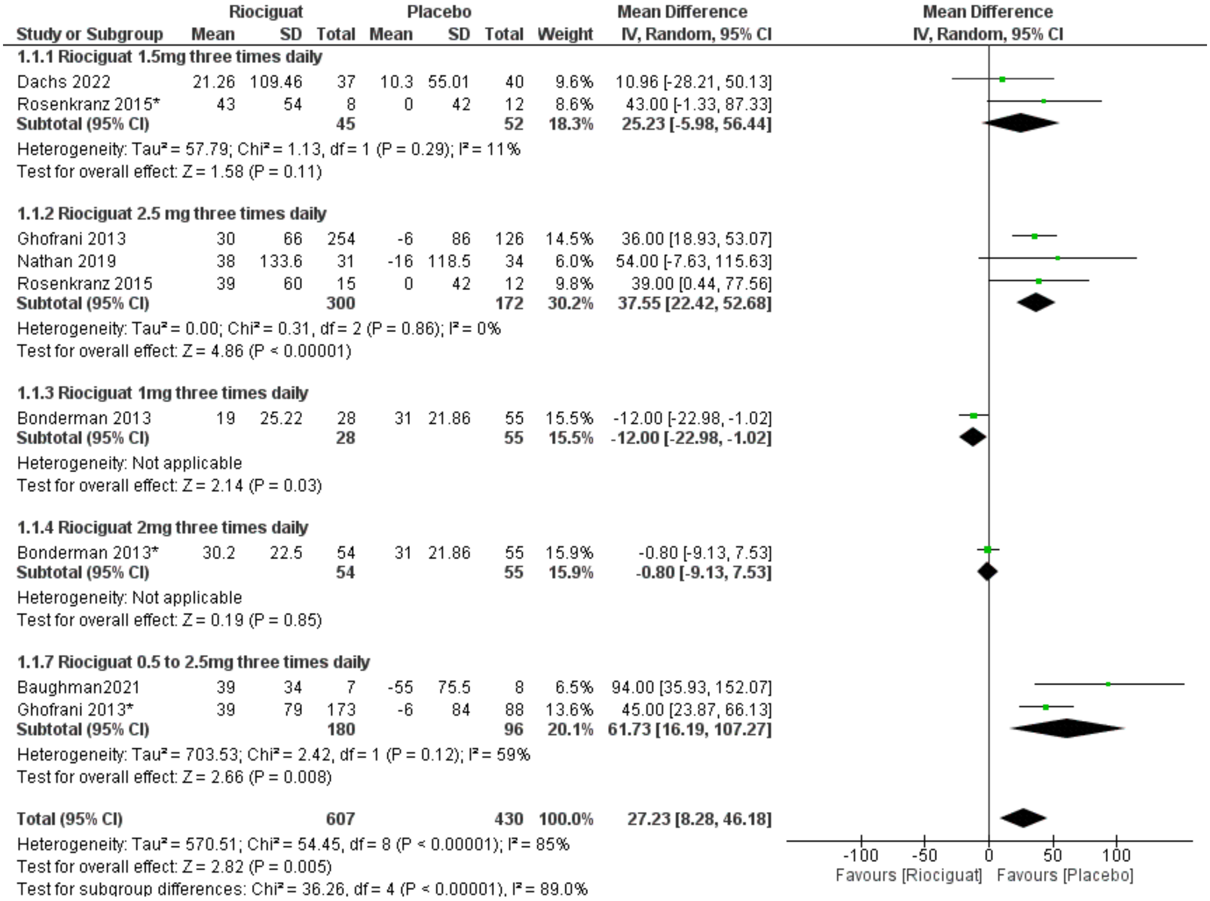

Figures S2 (B): Mean Pulmonary Artery Pressure (mPAP)

	Riociguat			Placebo				Mean Difference
**Study or Subgroup**	**Mean**	**SD**	**Total**	**Mean**	**SD**	**Total**	**Weight**	**IV, Random, 95% CI**
**1.2.1 Riociguat 2.5mg 3 times daily**								
Galie 2016	-4.4	8	115	-0.3	12	56	8.5%	-4.10 [-7.57, -0.63]
Galie 2016*	-3.5	8	119	-0.6	6	53	10.0%	-2.90 [-5.06, -0.74]
Ghofrani 2013	-4	8	235	-0.5	9.4	109	10.2%	-3.50 [-5.54, -1.46]
Rosenkranz 2015	0	0	0	0	0	0		Not estimable
**Subtotal (95% CI)**			**469**			**218**	**28.6%**	**-3.35 [-4.72, -1.99]**
Heterogeneity: Tau² = 0.00; Chi² = 0.37, df = 2 (P = 0.83); I² = 0%								
Test for overall effect: Z = 4.82 (P < 0.00001)								
**1.2.2 Riociguat 1.5 mg 3 three times daily**								
Dachs 2022	-2.69	7.726	39	-0.38	6.25	48	9.0%	-2.31 [-5.31, 0.69]
Rosenkranz 2015*	-3	10	7	1	8	11	3.5%	-4.00 [-12.79, 4.79]
**Subtotal (95% CI)**			**46**			**59**	**12.6%**	**-2.49 [-5.33, 0.35]**
Heterogeneity: Tau² = 0.00; Chi² = 0.13, df = 1 (P = 0.72); I² = 0%								
Test for overall effect: Z = 1.72 (P = 0.09)								
**1.2.3 Riociguat 0.5 to 2.5mg three times daily**								
Ghofrani 2013*	-4	7	156	0.8	7.3	84	10.3%	-4.80 [-6.71, -2.89]
Kim 2016	-4	7	109	1	7	65	10.0%	-5.00 [-7.15, -2.85]
Kim 2016*	-5	6	47	1	7	19	8.3%	-6.00 [-9.58, -2.42]
**Subtotal (95% CI)**			**312**			**168**	**28.6%**	**-5.04 [-6.37, -3.71]**
Heterogeneity: Tau² = 0.00; Chi² = 0.34, df = 2 (P = 0.84); I² = 0%								
Test for overall effect: Z = 7.45 (P < 0.00001)								
**1.2.4 Riociguat 1mg three times daily**								
Bonderman 2013	-0.7	2.8	28	-4	1.8	56	11.0%	3.30 [2.16, 4.44]
**Subtotal (95% CI)**			**28**			**56**	**11.0%**	**3.30 [2.16, 4.44]**
Heterogeneity: Not applicable								
Test for overall effect: Z = 5.68 (P < 0.00001)								
**1.2.5 Riociguat 2 mg three times daily**								
Bonderman 2013*	-6.1	2.1	54	-4	1.8	56	11.2%	-2.10 [-2.83, -1.37]
**Subtotal (95% CI)**			**54**			**56**	**11.2%**	**-2.10 [-2.83, -1.37]**
Heterogeneity: Not applicable								
Test for overall effect: Z = 5.62 (P < 0.00001)								
**1.2.6 Riociguat 2mg one time daily**								
Bonderman 2014	-5.1	4.7	10	-6.3	4.2	11	8.0%	1.20 [-2.63, 5.03]
**Subtotal (95% CI)**			**10**			**11**	**8.0%**	**1.20 [-2.63, 5.03]**
Heterogeneity: Not applicable								
Test for overall effect: Z = 0.61 (P = 0.54)								
**Total (95% CI)**			**919**			**568**	**100.0%**	**-2.61 [-4.60, -0.63]**
Heterogeneity: Tau² = 9.03; Chi² = 108.32, df = 10 (P < 0.00001); I² = 91%								
Test for overall effect: Z = 2.58 (P = 0.010)								
Test for subgroup differences: Chi² = 107.49, df = 5 (P < 0.00001), I² = 95.3%								

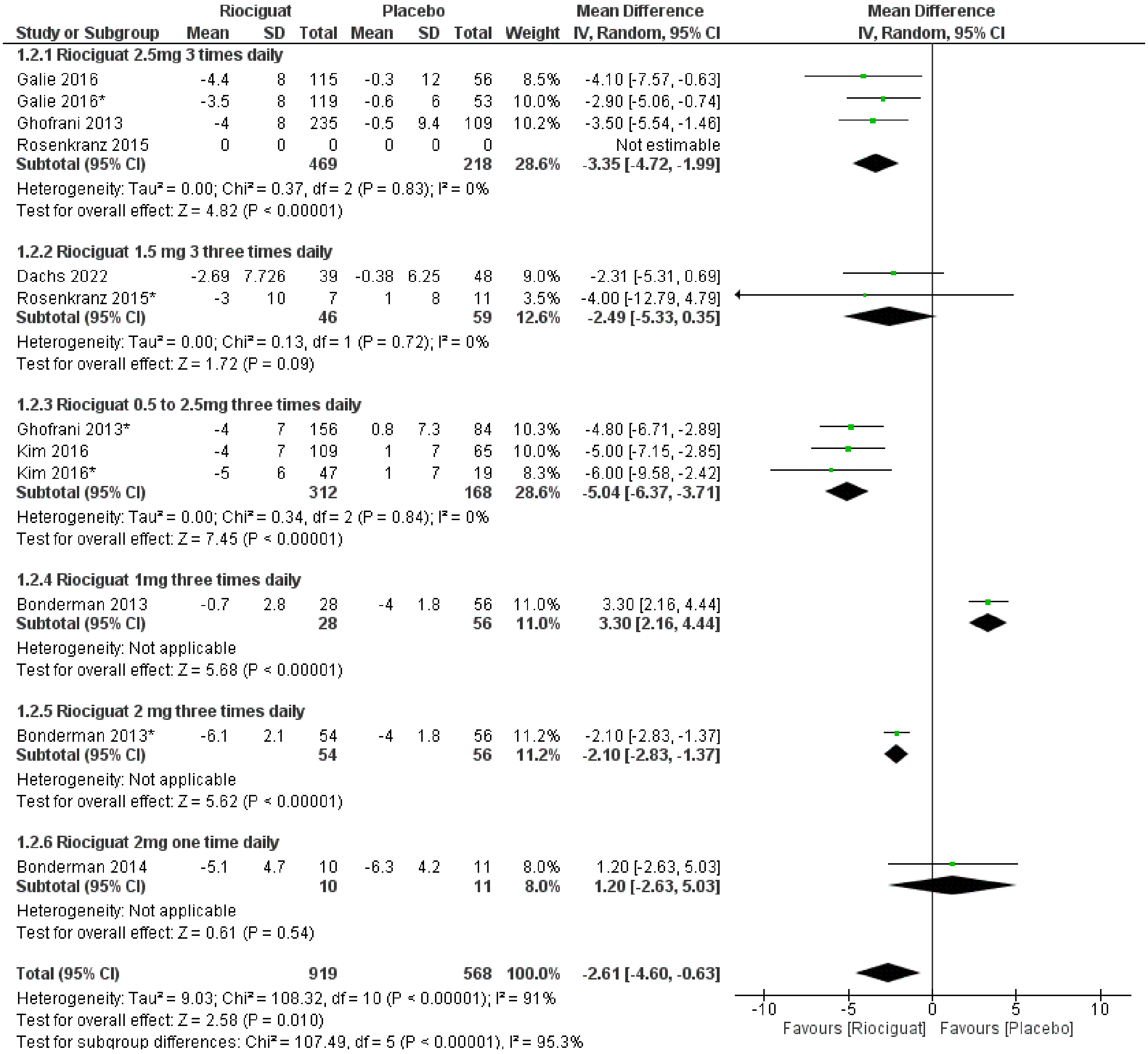

Figures S2 (C): Pulmonary Vascular Resistance

	Riociguat			Placebo				Mean Difference
**Study or Subgroup**	**Mean**	**SD**	**Total**	**Mean**	**SD**	**Total**	**Weight**	**IV, Random, 95% CI**
**1.3.1 Riociguat 2.5mg three times daily**								
Galie 2016	-259	296	115	17	361	55	4.9%	-276.00 [-385.68, -166.32]
Galie 2016*	-188	215	117	-36	263	52	7.2%	-152.00 [-233.41, -70.59]
Ghofrani 2013	-223	260	232	-9	317	107	8.6%	-214.00 [-282.75, -145.25]
Rosenkranz 2015	-250	10	13	-66	632	11	0.6%	-184.00 [-557.52, 189.52]
**Subtotal (95% CI)**			**477**			**225**	**21.3%**	**-204.85 [-255.84, -153.87]**
Heterogeneity: Tau² = 284.50; Chi² = 3.31, df = 3 (P = 0.35); I² = 10%								
Test for overall effect: Z = 7.87 (P < 0.00001)								
**1.3.2 Riociguat 1.5mg three times daily**								
Dachs 2022	-65.26	266.7	35	7.14	265.2	46	4.5%	-72.40 [-189.36, 44.56]
Rosenkranz 2015*	-126	368	7	-66	632	1	0.1%	-60.00 [-1328.34, 1208.34]
**Subtotal (95% CI)**			**42**			**47**	**4.5%**	**-72.30 [-188.76, 44.17]**
Heterogeneity: Tau² = 0.00; Chi² = 0.00, df = 1 (P = 0.98); I² = 0%								
Test for overall effect: Z = 1.22 (P = 0.22)								
**1.3.4 Riociguat 0.5 to 2.5mg three times daily**								
Baughman2021	4.4	1.19	8	5.1	2.1	8	16.6%	-0.70 [-2.37, 0.97]
Ghofrani 2013*	-226	248	15	23	274	82	3.5%	-249.00 [-387.81, -110.19]
Kim 2016	-257	279	105	33	292	63	6.4%	-290.00 [-379.70, -200.30]
Kim 2016*	-154	127	46	-11	205	19	5.6%	-143.00 [-242.22, -43.78]
**Subtotal (95% CI)**			**174**			**172**	**32.1%**	**-165.63 [-332.75, 1.50]**
Heterogeneity: Tau² = 26773.99; Chi² = 60.11, df = 3 (P < 0.00001); I² = 95%								
Test for overall effect: Z = 1.94 (P = 0.05)								
**1.3.5 Riociguat 1mg three times daily**								
Bonderman 2013	-33.1	28.36	56	-37	28.36	56	16.2%	3.90 [-6.60, 14.40]
**Subtotal (95% CI)**			**56**			**56**	**16.2%**	**3.90 [-6.60, 14.40]**
Heterogeneity: Not applicable								
Test for overall effect: Z = 0.73 (P = 0.47)								
**1.3.6 Riociguat 2mg one time daily**								
Bonderman 2014	-20.8	69.75	10	-4.6	69.55	11	9.7%	-16.20 [-75.85, 43.45]
**Subtotal (95% CI)**			**10**			**11**	**9.7%**	**-16.20 [-75.85, 43.45]**
Heterogeneity: Not applicable								
Test for overall effect: Z = 0.53 (P = 0.59)								
**1.3.7 Riociguat 2mg three times daily**								
Bonderman 2013*	-78.2	35.5	54	-37	28.3	56	16.1%	-41.20 [-53.22, -29.18]
**Subtotal (95% CI)**			**54**			**56**	**16.1%**	**-41.20 [-53.22, -29.18]**
Heterogeneity: Not applicable								
Test for overall effect: Z = 6.72 (P < 0.00001)								
**Total (95% CI)**			**813**			**567**	**100.0%**	**-90.27 [-119.30, -61.23]**
Heterogeneity: Tau² = 1324.16; Chi² = 179.88, df = 12 (P < 0.00001); I² = 93%								
Test for overall effect: Z = 6.09 (P < 0.00001)								
Test for subgroup differences: Chi² = 86.01, df = 5 (P < 0.00001), I² = 94.2%								

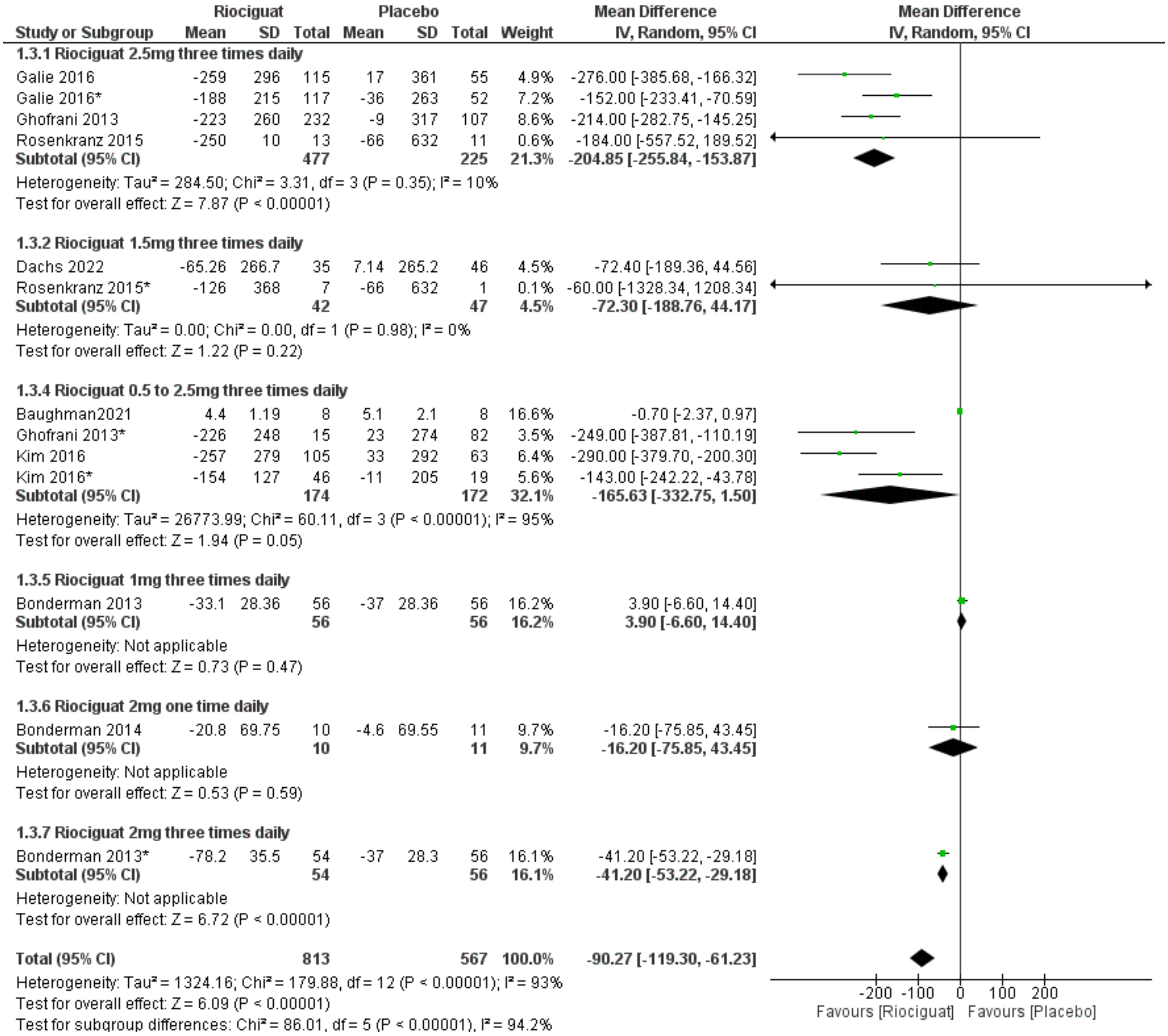

Figures S2 (D): Right Atrial Pressure (RAP)

Study or Subgroup	Riociguat Mean	Riociguat SD	Riociguat Total	Placebo Mean	Placebo SD	Placebo Total	Weight	Mean Difference IV, Random, 95% CI
**1.4.1 Riociguat 2.5mg three times daily**								
Galie 2016	-0.2	7	116	1.7	5.7	55	6.9%	-1.90 [-3.87, 0.07]
Galie 2016*	-0.2	4.3	119	0.2	3.8	53	9.8%	-0.40 [-1.68, 0.88]
Ghofrani 2013	-0.2	5.8	235	1	5	108	10.2%	-1.20 [-2.40, -0.00]
Rosenkranz 2015	-0.3	4.3	14	2.7	6.4	10	2.2%	-3.00 [-7.56, 1.56]
**Subtotal (95% CI)**			**484**			**226**	**29.1%**	**-1.06 [-1.85, -0.27]**
Heterogeneity: $Tau^2 = 0.00$; $Chi^2 = 2.46$, df = 3 (P = 0.48); $I^2 = 0\%$								
Test for overall effect: Z = 2.64 (P = 0.008)								
**1.4.2 Riociguat 1.5mg three times daily**								
Dachs 2022	0.21	6.502	38	-0.98	4.128	48	5.6%	1.19 [-1.18, 3.56]
Rosenkranz 2015*	-1.7	2.8	7	2.7	6.4	10	2.2%	-4.40 [-8.88, 0.08]
**Subtotal (95% CI)**			**45**			**58**	**7.9%**	**-1.27 [-6.71, 4.17]**
Heterogeneity: $Tau^2 = 12.28$; $Chi^2 = 4.68$, df = 1 (P = 0.03); $I^2 = 79\%$								
Test for overall effect: Z = 0.46 (P = 0.65)								
**1.4.3 Riociguat 1mg three times daily**								
Bonderman 2013	0.6	1.63	28	-1.1	1.06	56	12.6%	1.70 [1.04, 2.36]
**Subtotal (95% CI)**			**28**			**56**	**12.6%**	**1.70 [1.04, 2.36]**
Heterogeneity: Not applicable								
Test for overall effect: Z = 5.01 (P < 0.00001)								
**1.4.4 Riociguat 0.5mg to 2.5mg three times daily**								
Baughman2021	4.4	1.19	8	5.1	2.1	8	8.1%	-0.70 [-2.37, 0.97]
Ghofrani 2013*	-1	5	157	-0.6	5.2	84	9.4%	-0.40 [-1.76, 0.96]
Kim 2016	-0.8	5.4	110	-0.7	5.6	65	8.0%	-0.10 [-1.79, 1.59]
Kim 2016*	-1.6	4.5	47	0.1	3.3	19	7.0%	-1.70 [-3.66, 0.26]
**Subtotal (95% CI)**			**322**			**176**	**32.5%**	**-0.63 [-1.44, 0.19]**
Heterogeneity: $Tau^2 = 0.00$; $Chi^2 = 1.63$, df = 3 (P = 0.65); $I^2 = 0\%$								
Test for overall effect: Z = 1.50 (P = 0.13)								
**1.4.5 Riociguat 2mg one time daily**								
Bonderman 2014	-0.9	3.18	11	-1.7	3.48	10	4.4%	0.80 [-2.06, 3.66]
**Subtotal (95% CI)**			**11**			**10**	**4.4%**	**0.80 [-2.06, 3.66]**
Heterogeneity: Not applicable								
Test for overall effect: Z = 0.55 (P = 0.58)								
**1.4.6 Riociguat 2mg three times daily**								
Bonderman 2013*	-0.6	0.99	54	-1.1	1.06	56	13.6%	0.50 [0.12, 0.88]
**Subtotal (95% CI)**			**54**			**56**	**13.6%**	**0.50 [0.12, 0.88]**
Heterogeneity: Not applicable								
Test for overall effect: Z = 2.56 (P = 0.01)								
**Total (95% CI)**			**944**			**582**	**100.0%**	**-0.29 [-1.02, 0.44]**
Heterogeneity: $Tau^2 = 0.98$; $Chi^2 = 43.25$, df = 12 (P < 0.0001); $I^2 = 72\%$								
Test for overall effect: Z = 0.79 (P = 0.43)								
Test for subgroup differences: $Chi^2 = 34.69$, df = 5 (P < 0.00001), $I^2 = 85.6\%$								

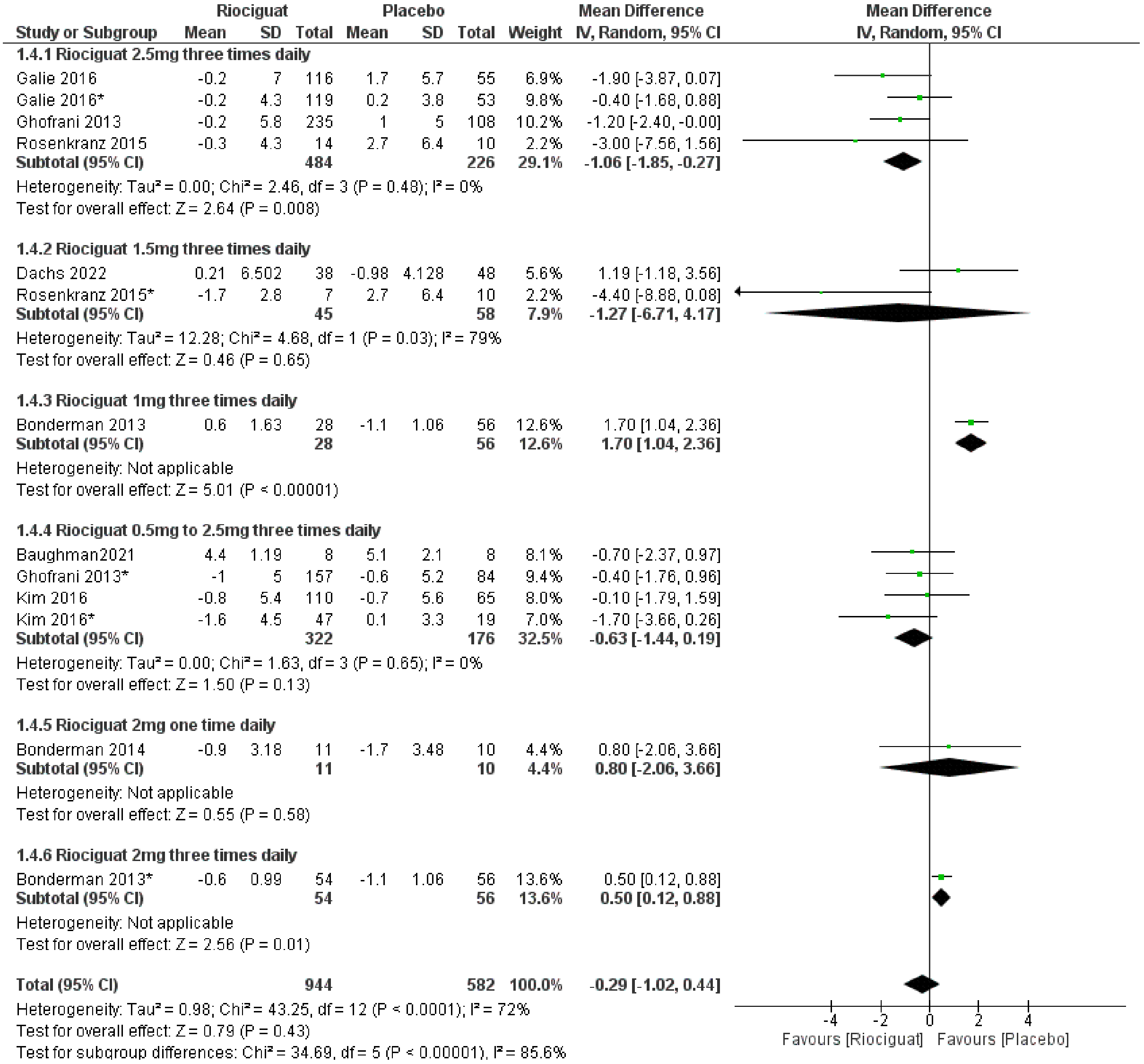

Figures S2 (E): Mean Arterial Pressure (MAP)

	Riociguat			Placebo				Mean Difference
Study or Subgroup	Mean	SD	Total	Mean	SD	Total	Weight	IV, Random, 95% CI
**1.5.1 Riociguat 2.5mg three times daily**								
Galie 2016	-10	10	113	-1.3	13	57	16.2%	-8.70 [-12.55, -4.85]
Galie 2016*	-7.6	12	116	-1.5	12	52	16.1%	-6.10 [-10.03, -2.17]
Ghofrani 2013	-9	11	229	-1	13	109	16.9%	-8.00 [-10.83, -5.17]
**Subtotal (95% CI)**			**458**			**218**	**49.2%**	**-7.71 [-9.67, -5.74]**
Heterogeneity: Tau^2 = 0.00; Chi^2 = 0.94, df = 2 (P = 0.62); I^2 = 0%								
Test for overall effect: Z = 7.67 (P < 0.00001)								
**1.5.3 Riociguat 0.5 to 2.5mg three times daily**								
Ghofrani 2013*	-0.3	11.8	78	-9	12	155	16.6%	8.70 [5.47, 11.93]
Kim 2016	-10	13	108	-0.4	11	61	16.3%	-9.60 [-13.29, -5.91]
**Subtotal (95% CI)**			**186**			**216**	**32.9%**	**-0.43 [-18.36, 17.51]**
Heterogeneity: Tau^2 = 164.31; Chi^2 = 53.47, df = 1 (P < 0.00001); I^2 = 98%								
Test for overall effect: Z = 0.05 (P = 0.96)								
**1.5.4 Riociguat 1mg three times daily**								
Bonderman 2013	0.2	0.14	28	0	0	56		Not estimable
**Subtotal (95% CI)**			**28**			**56**		**Not estimable**
Heterogeneity: Not applicable								
Test for overall effect: Not applicable								
**1.5.5 Riociguat 2mg one time daily**								
Bonderman 2014	0.5	0.316	10	0.04	0.33	11	17.9%	0.46 [0.18, 0.74]
**Subtotal (95% CI)**			**10**			**11**	**17.9%**	**0.46 [0.18, 0.74]**
Heterogeneity: Not applicable								
Test for overall effect: Z = 3.26 (P = 0.001)								
**1.5.6 Riociguat 2mg three times daily**								
Bonderman 2013*	0.4	0.14	54	0	0	56		Not estimable
**Subtotal (95% CI)**			**54**			**56**		**Not estimable**
Heterogeneity: Not applicable								
Test for overall effect: Not applicable								
**Total (95% CI)**			**736**			**557**	**100.0%**	**-3.77 [-8.72, 1.17]**
Heterogeneity: Tau^2 = 35.52; Chi^2 = 119.29, df = 5 (P < 0.00001); I^2 = 96%								
Test for overall effect: Z = 1.50 (P = 0.13)								
Test for subgroup differences: Chi^2 = 64.74, df = 2 (P < 0.00001), I^2 = 96.9%								

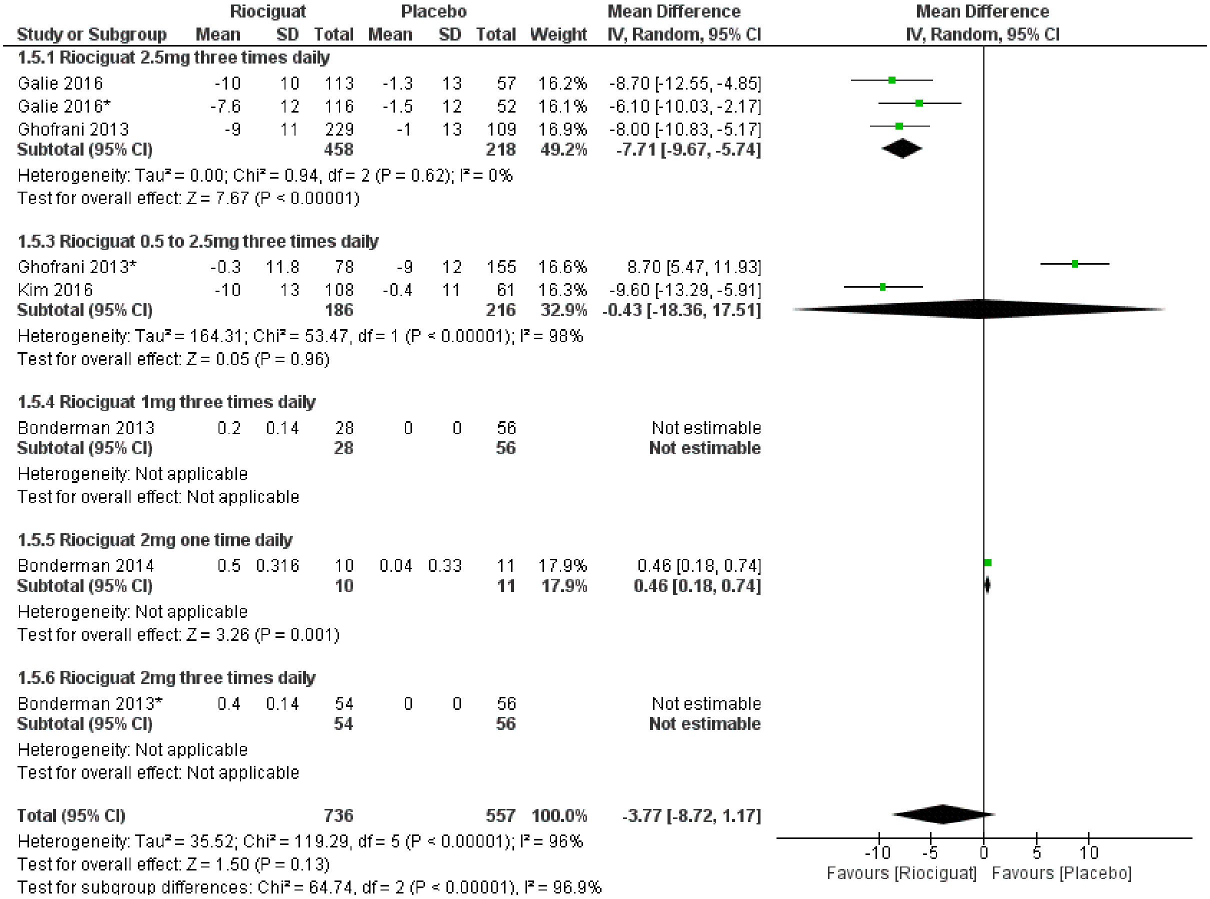

Figures S2 (F): Cardiac Index

Study or Subgroup	Riociguat Mean	Riociguat SD	Riociguat Total	Placebo Mean	Placebo SD	Placebo Total	Weight	Mean Difference IV, Random, 95% CI
**1.6.1 Riociguat 2.5mg three times daily**								
Galie 2016	0.6	0.6	115	-0.1	0.7	55	13.4%	0.70 [0.48, 0.92]
Galie 2016*	0.5	0.6	118	0.04	0.5	53	14.5%	0.46 [0.29, 0.63]
Nathan 2019	2.6	0.7	73	2.6	0.7	74	13.1%	0.00 [-0.23, 0.23]
Rosenkranz 2015	0.4	0.6	13	0.2	0.7	11	6.6%	0.20 [-0.33, 0.73]
**Subtotal (95% CI)**			**319**			**193**	**47.6%**	**0.36 [0.04, 0.68]**
Heterogeneity: Tau² = 0.09; Chi² = 20.49, df = 3 (P = 0.0001); I² = 85%								
Test for overall effect: Z = 2.18 (P = 0.03)								
**1.6.2 Riociguat 0.5 to 2.5 mg three times daily**								
Kim 2016	0.5	0.6	108	-0.1	0.6	64	14.2%	0.60 [0.41, 0.79]
Kim 2016*	0.4	0.5	47	0.2	0.5	19	12.1%	0.20 [-0.07, 0.47]
**Subtotal (95% CI)**			**155**			**83**	**26.2%**	**0.41 [0.02, 0.80]**
Heterogeneity: Tau² = 0.07; Chi² = 5.83, df = 1 (P = 0.02); I² = 83%								
Test for overall effect: Z = 2.06 (P = 0.04)								
**1.6.3 Riociguat 1mg three times daily**								
Bonderman 2013	0.2	0.14	28	0	0	56		Not estimable
**Subtotal (95% CI)**			**28**			**56**		**Not estimable**
Heterogeneity: Not applicable								
Test for overall effect: Not applicable								
**1.6.4 Riociguat 2mg one time daily**								
Bonderman 2014	0.5	0.316	10	0.04	0.33	11	11.8%	0.46 [0.18, 0.74]
**Subtotal (95% CI)**			**10**			**11**	**11.8%**	**0.46 [0.18, 0.74]**
Heterogeneity: Not applicable								
Test for overall effect: Z = 3.26 (P = 0.001)								
**1.6.5 Riociguat 1.5mg three times daily**								
Dachs 2022	0.14	0.707	35	-0.03	0.471	46	12.0%	0.17 [-0.10, 0.44]
Rosenkranz 2015*	0.3	1.3	7	0.2	0.7	11	2.3%	0.10 [-0.95, 1.15]
**Subtotal (95% CI)**			**42**			**57**	**14.3%**	**0.17 [-0.10, 0.43]**
Heterogeneity: Tau² = 0.00; Chi² = 0.02, df = 1 (P = 0.90); I² = 0%								
Test for overall effect: Z = 1.24 (P = 0.22)								
**1.6.6 Riociguat 2mg three times daily**								
Bonderman 2013*	0.4	0.14	54	0	0	56		Not estimable
**Subtotal (95% CI)**			**54**			**56**		**Not estimable**
Heterogeneity: Not applicable								
Test for overall effect: Not applicable								
**Total (95% CI)**			**608**			**456**	**100.0%**	**0.36 [0.19, 0.53]**
Heterogeneity: Tau² = 0.05; Chi² = 30.38, df = 8 (P = 0.0002); I² = 74%								
Test for overall effect: Z = 4.07 (P < 0.0001)								
Test for subgroup differences: Chi² = 2.55, df = 3 (P = 0.47), I² = 0%								

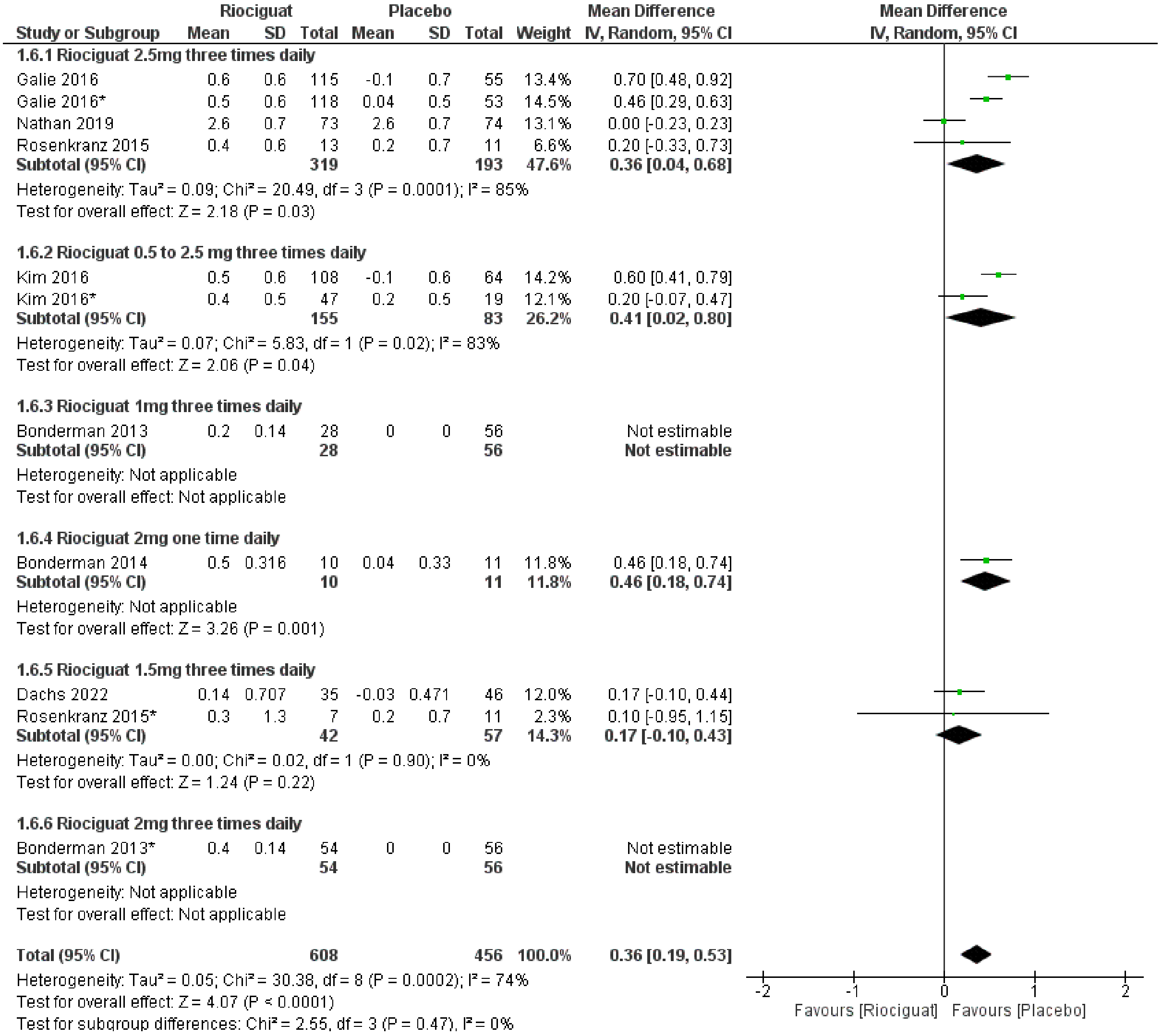

Figures S2 (G): Cardiac Output

Study or Subgroup	Riociguat Mean	Riociguat SD	Riociguat Total	Placebo Mean	Placebo SD	Placebo Total	Weight	Mean Difference IV, Random, 95% CI
**1.7.1 Riociguat 2.5mg three times daily**								
Galie 2016	1	1	115	-0.1	1.2	55	12.6%	1.10 [0.73, 1.47]
Galie 2016*	0.9	1	118	0.1	0.9	53	16.8%	0.80 [0.50, 1.10]
Ghofrani 2013	1	1	233	-0.01	1.07	108	23.0%	1.01 [0.77, 1.25]
**Subtotal (95% CI)**			**466**			**216**	**52.5%**	**0.96 [0.80, 1.13]**
Heterogeneity: Tau² = 0.00; Chi² = 1.80, df = 2 (P = 0.41); I² = 0%								
Test for overall effect: Z = 11.33 (P < 0.00001)								
**1.7.2 Riociguat 0.5 to 2.5mg three times daily**								
Ghofrani 2013*	0.8	1.1	155	-0.03	1.07	83	18.0%	0.83 [0.54, 1.12]
Kim 2016	0.8	1.1	108	-0.1	1.1	64	14.1%	0.90 [0.56, 1.24]
Kim 2016*	0.7	1.1	47	0.3	0.9	19	7.2%	0.40 [-0.11, 0.91]
**Subtotal (95% CI)**			**310**			**166**	**39.3%**	**0.77 [0.53, 1.02]**
Heterogeneity: Tau² = 0.01; Chi² = 2.70, df = 2 (P = 0.26); I² = 26%								
Test for overall effect: Z = 6.27 (P < 0.00001)								
**1.7.3 Riociguat 1.5mg three times daily**								
Dachs 2022	0.37	1.263	38	-0.11	0.921	48	8.1%	0.48 [0.00, 0.96]
**Subtotal (95% CI)**			**38**			**48**	**8.1%**	**0.48 [0.00, 0.96]**
Heterogeneity: Not applicable								
Test for overall effect: Z = 1.97 (P = 0.05)								
**1.7.6 Riociguat 2mg one time daily**								
Bonderman 2014	1	6.3	10	0.08	0.66	11	0.1%	0.92 [-3.00, 4.84]
**Subtotal (95% CI)**			**10**			**11**	**0.1%**	**0.92 [-3.00, 4.84]**
Heterogeneity: Not applicable								
Test for overall effect: Z = 0.46 (P = 0.65)								
**Total (95% CI)**			**824**			**441**	**100.0%**	**0.85 [0.70, 1.00]**
Heterogeneity: Tau² = 0.01; Chi² = 8.92, df = 7 (P = 0.26); I² = 22%								
Test for overall effect: Z = 11.40 (P < 0.00001)								
Test for subgroup differences: Chi² = 4.39, df = 3 (P = 0.22), I² = 31.7%								

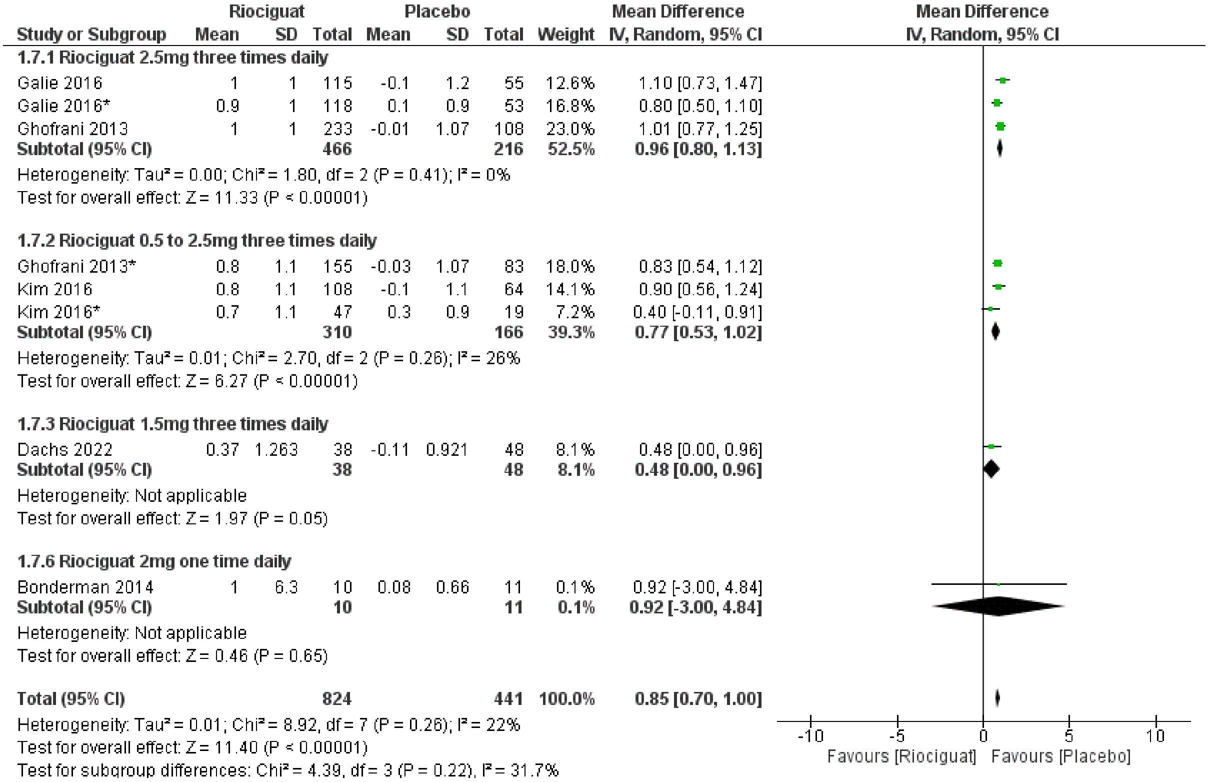

Figures S2 (G): Cardiac Output

Study or Subgroup	Riociguat Mean	Riociguat SD	Riociguat Total	Placebo Mean	Placebo SD	Placebo Total	Weight	Mean Difference IV, Random, 95% CI
**1.7.1 Riociguat 2.5mg three times daily**								
Galie 2016	1	1	115	-0.1	1.2	55	12.6%	1.10 [0.73, 1.47]
Galie 2016*	0.9	1	118	0.1	0.9	53	16.8%	0.80 [0.50, 1.10]
Ghofrani 2013	1	1	233	-0.01	1.07	108	23.0%	1.01 [0.77, 1.25]
**Subtotal (95% CI)**			**466**			**216**	**52.5%**	**0.96 [0.80, 1.13]**
Heterogeneity: Tau² = 0.00; Chi² = 1.80, df = 2 (P = 0.41); I² = 0%								
Test for overall effect: Z = 11.33 (P < 0.00001)								
**1.7.2 Riociguat 0.5 to 2.5mg three times daily**								
Ghofrani 2013*	0.8	1.1	155	-0.03	1.07	83	18.0%	0.83 [0.54, 1.12]
Kim 2016	0.8	1.1	108	-0.1	1.1	64	14.1%	0.90 [0.56, 1.24]
Kim 2016*	0.7	1.1	47	0.3	0.9	19	7.2%	0.40 [-0.11, 0.91]
**Subtotal (95% CI)**			**310**			**166**	**39.3%**	**0.77 [0.53, 1.02]**
Heterogeneity: Tau² = 0.01; Chi² = 2.70, df = 2 (P = 0.26); I² = 26%								
Test for overall effect: Z = 6.27 (P < 0.00001)								
**1.7.3 Riociguat 1.5mg three times daily**								
Dachs 2022	0.37	1.263	38	-0.11	0.921	48	8.1%	0.48 [0.00, 0.96]
**Subtotal (95% CI)**			**38**			**48**	**8.1%**	**0.48 [0.00, 0.96]**
Heterogeneity: Not applicable								
Test for overall effect: Z = 1.97 (P = 0.05)								
**1.7.6 Riociguat 2mg one time daily**								
Bonderman 2014	1	6.3	10	0.08	0.66	11	0.1%	0.92 [-3.00, 4.84]
**Subtotal (95% CI)**			**10**			**11**	**0.1%**	**0.92 [-3.00, 4.84]**
Heterogeneity: Not applicable								
Test for overall effect: Z = 0.46 (P = 0.65)								
**Total (95% CI)**			**824**			**441**	**100.0%**	**0.85 [0.70, 1.00]**
Heterogeneity: Tau² = 0.01; Chi² = 8.92, df = 7 (P = 0.26); I² = 22%								
Test for overall effect: Z = 11.40 (P < 0.00001)								
Test for subgroup differences: Chi² = 4.39, df = 3 (P = 0.22), I² = 31.7%								

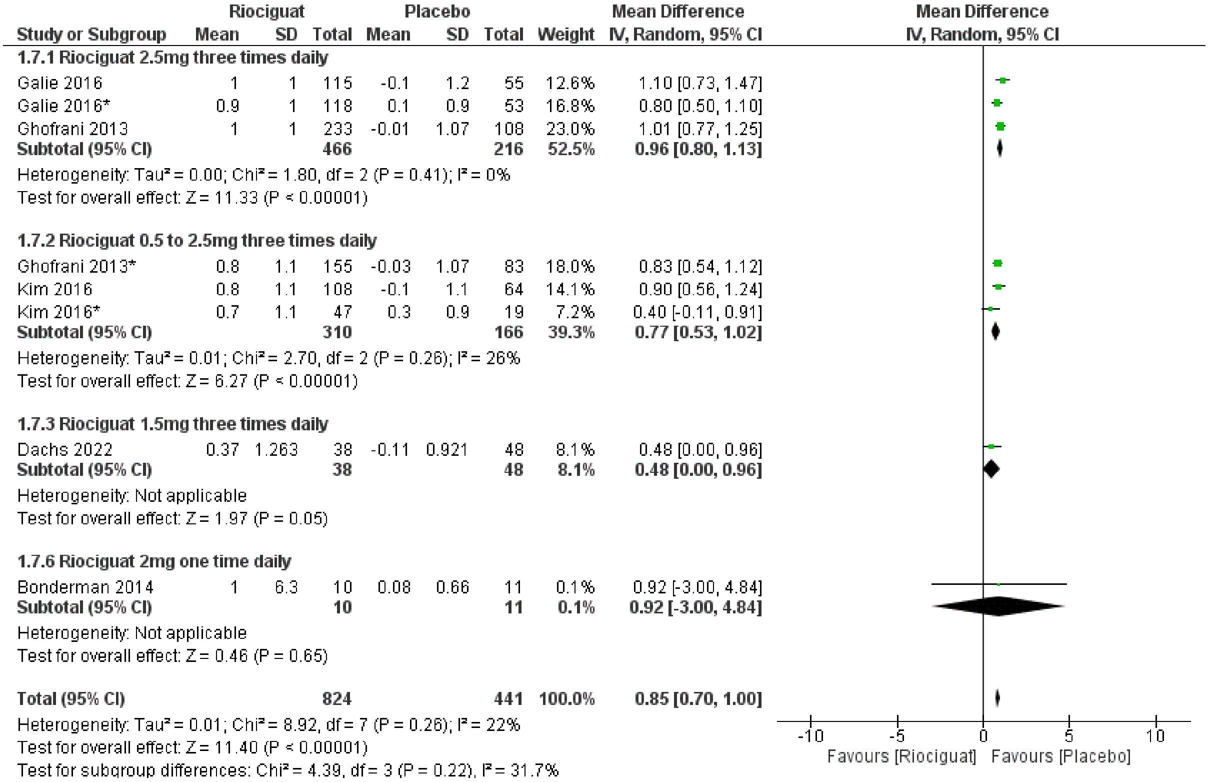

Figures S2 (H): N-terminal pro-type B natriuretic peptide (NT-proBNP)

Study or Subgroup	Riociguat Mean	Riociguat SD	Riociguat Total	Placebo Mean	Placebo SD	Placebo Total	Weight	Mean Difference IV, Random, 95% CI
**1.8.1 Riociguat 2..5mg three times daily**								
Ghofrani 2013	-198	1,721	228	232	1,011	106	17.1%	-430.00 [-724.86, -135.14]
Rosenkranz 2015	-164	317	13	-872	1,147	7	5.6%	708.00 [-158.99, 1574.99]
**Subtotal (95% CI)**			**241**			**113**	**22.7%**	**62.97 [-1042.25, 1168.19]**
Heterogeneity: Tau² = 538368.38; Chi² = 5.93, df = 1 (P = 0.01); I² = 83%								
Test for overall effect: Z = 0.11 (P = 0.91)								
**1.8.2 Riociguat 0.5 to 2.5mg three times daily**								
Ghofrani 2013*	-291	1,717	150	76	1,447	73	13.1%	-367.00 [-797.91, 63.91]
**Subtotal (95% CI)**			**150**			**73**	**13.1%**	**-367.00 [-797.91, 63.91]**
Heterogeneity: Not applicable								
Test for overall effect: Z = 1.67 (P = 0.10)								
**1.8.3 Riociguat 1.5mg three times daily**								
Dachs 2022	60.35	5.14	36	76	2.18	43	23.4%	-15.65 [-17.45, -13.85]
Rosenkranz 2015*	-872	1,147	7	-46	697	12	5.0%	-826.00 [-1762.75, 110.75]
**Subtotal (95% CI)**			**43**			**55**	**28.4%**	**-279.88 [-1024.41, 464.65]**
Heterogeneity: Tau² = 214119.03; Chi² = 2.87, df = 1 (P = 0.09); I² = 65%								
Test for overall effect: Z = 0.74 (P = 0.46)								
**1.8.4 Riociguat 2mg three times daily**								
Bonderman 2013*	-175	446.55	52	171	725.24	56	19.3%	-346.00 [-571.41, -120.59]
**Subtotal (95% CI)**			**52**			**56**	**19.3%**	**-346.00 [-571.41, -120.59]**
Heterogeneity: Not applicable								
Test for overall effect: Z = 3.01 (P = 0.003)								
**1.8.5 Riociguat 1mg three times daily**								
Bonderman 2013	-213	683.86	28	171	725.24	56	16.5%	-384.00 [-700.61, -67.39]
**Subtotal (95% CI)**			**28**			**56**	**16.5%**	**-384.00 [-700.61, -67.39]**
Heterogeneity: Not applicable								
Test for overall effect: Z = 2.38 (P = 0.02)								
**Total (95% CI)**			**514**			**353**	**100.0%**	**-256.77 [-491.99, -21.55]**
Heterogeneity: Tau² = 61448.26; Chi² = 29.14, df = 6 (P < 0.0001); I² = 79%								
Test for overall effect: Z = 2.14 (P = 0.03)								
Test for subgroup differences: Chi² = 0.62, df = 4 (P = 0.96), I² = 0%								

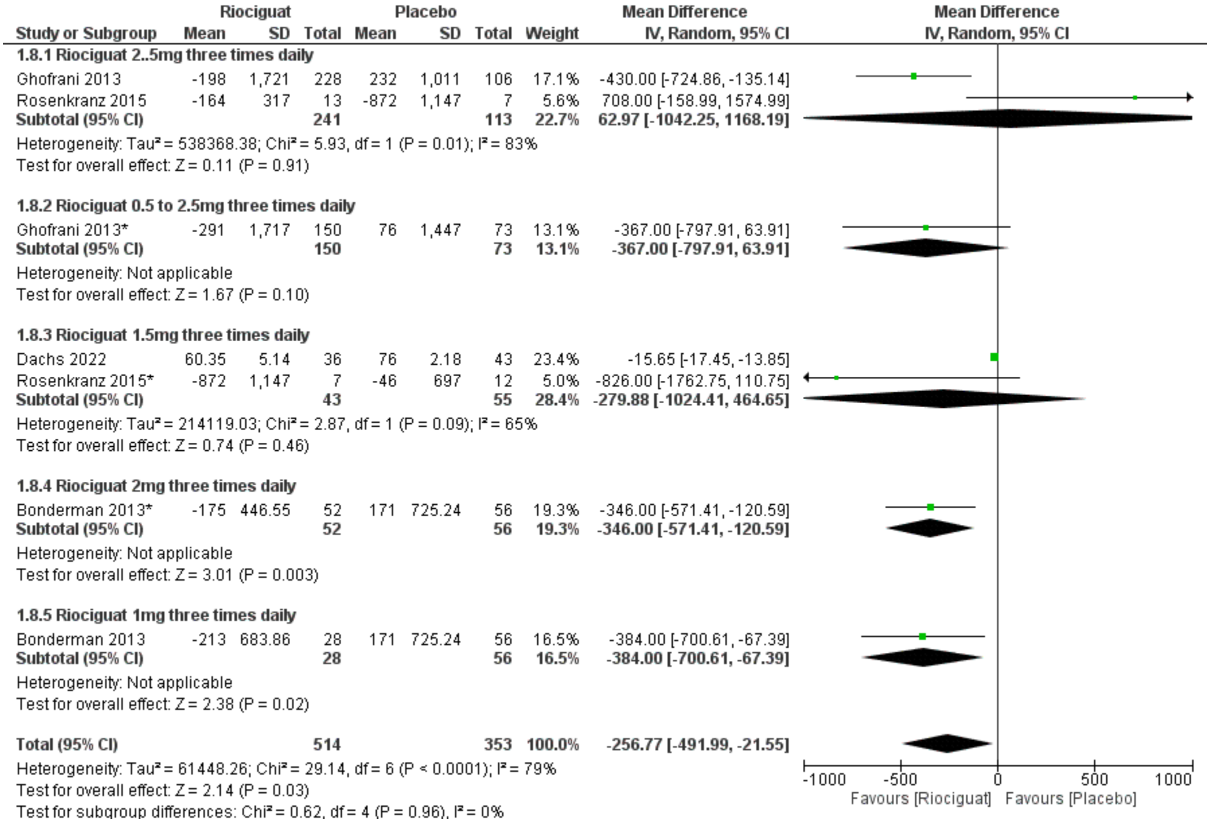

Figures S2 (I): Systemic Vascular Resistance

Study or Subgroup	Riociguat Mean	Riociguat SD	Riociguat Total	Placebo Mean	Placebo SD	Placebo Total	Weight	Mean Difference IV, Random, 95% CI
**1.9.1 Riociguat 0.5 to 2.5mg three times daily**								
Kim 2016	-537	559	106	56	615	60	13.7%	-593.00 [-781.52, -404.48]
Kim 2016*	-272	484	47	-121	551	17	9.8%	-151.00 [-447.23, 145.23]
**Subtotal (95% CI)**			**153**			**77**	**23.5%**	**-387.37 [-819.48, 44.73]**
Heterogeneity: Tau² = 81634.63; Chi² = 6.09, df = 1 (P = 0.01); I² = 84%								
Test for overall effect: Z = 1.76 (P = 0.08)								
**1.9.2 Riociguat 2.5mg three times daily**								
Galie 2016	-525	464	112	-56	597	54	14.0%	-469.00 [-649.94, -288.06]
Galie 2016*	-374	383	15	-79	410	52	12.4%	-295.00 [-518.57, -71.43]
**Subtotal (95% CI)**			**127**			**106**	**26.4%**	**-394.90 [-563.53, -226.27]**
Heterogeneity: Tau² = 4370.79; Chi² = 1.41, df = 1 (P = 0.24); I² = 29%								
Test for overall effect: Z = 4.59 (P < 0.00001)								
**1.9.3 Riociguat 1.5mg three times daily**								
Dachs 2022	-91.1	500	33	-54.9	392.1	41	12.9%	-36.20 [-244.78, 172.38]
**Subtotal (95% CI)**			**33**			**41**	**12.9%**	**-36.20 [-244.78, 172.38]**
Heterogeneity: Not applicable								
Test for overall effect: Z = 0.34 (P = 0.73)								
**1.9.4 Riociguat 1mg three times daily**								
Bonderman 2013	-123.8	100.52	28	-94.5	90.08	56	18.5%	-29.30 [-73.38, 14.78]
**Subtotal (95% CI)**			**28**			**56**	**18.5%**	**-29.30 [-73.38, 14.78]**
Heterogeneity: Not applicable								
Test for overall effect: Z = 1.30 (P = 0.19)								
**1.9.5 Riociguat 2mg three times daily**								
Bonderman 2013*	-33.12	74.06	54	-94.5	90.08	56	18.7%	61.38 [30.61, 92.15]
**Subtotal (95% CI)**			**54**			**56**	**18.7%**	**61.38 [30.61, 92.15]**
Heterogeneity: Not applicable								
Test for overall effect: Z = 3.91 (P < 0.0001)								
**Total (95% CI)**			**395**			**336**	**100.0%**	**-197.16 [-331.28, -63.03]**
Heterogeneity: Tau² = 24857.49; Chi² = 88.36, df = 6 (P < 0.00001); I² = 93%								
Test for overall effect: Z = 2.88 (P = 0.004)								
Test for subgroup differences: Chi² = 38.70, df = 4 (P < 0.00001), I² = 89.7%								

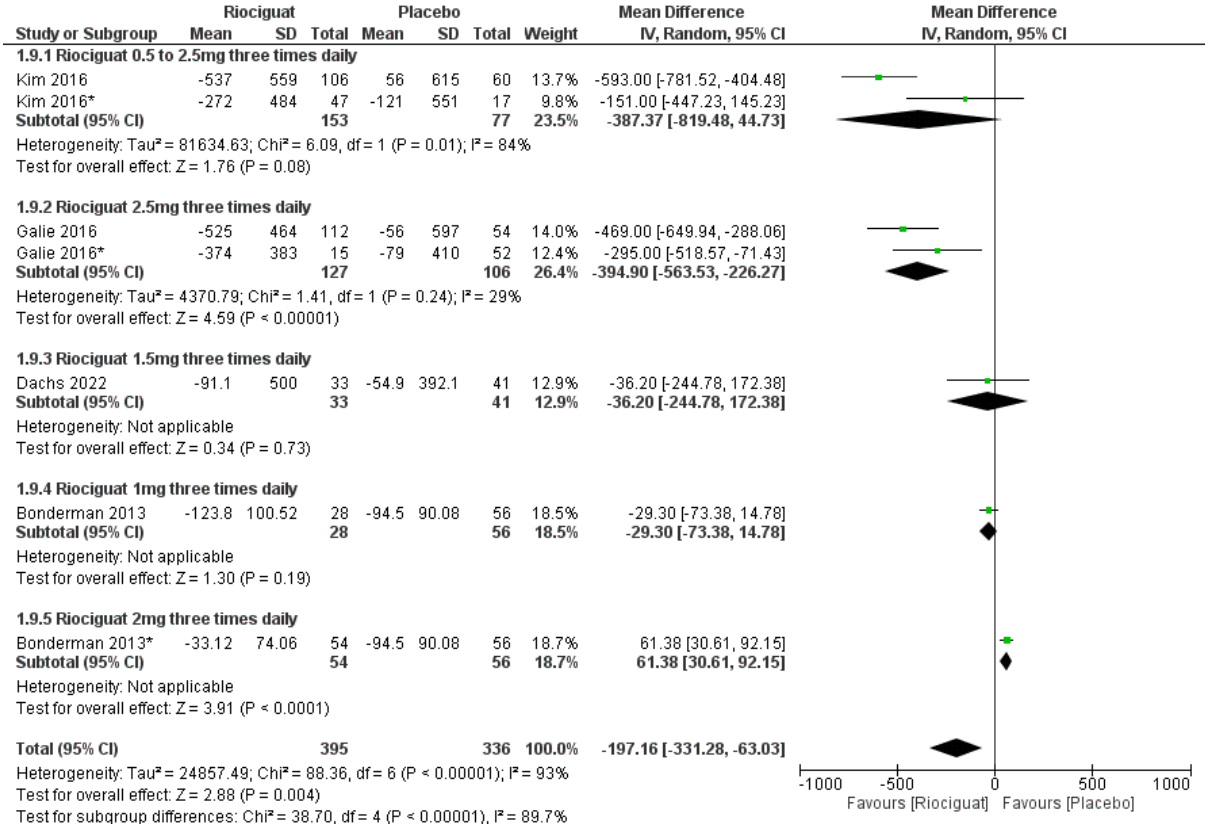

Figures S2 (J): Pulmonary Artery Wedge Pressure

	Riociguat			Placebo				Mean Difference
Study or Subgroup	Mean	SD	Total	Mean	SD	Total	Weight	IV, Random, 95% CI
**1.10.1 Riociguat 2.5mg three times daily**								
Ghofrani 2013	1	1.4	234	0.5	4.7	108	14.4%	0.50 [-0.40, 1.40]
Nathan 2019	11	3	73	11	3	74	14.2%	0.00 [-0.97, 0.97]
**Subtotal (95% CI)**			**307**			**182**	**28.6%**	**0.27 [-0.39, 0.93]**
Heterogeneity: Tau² = 0.00; Chi² = 0.55, df = 1 (P = 0.46); I² = 0%								
Test for overall effect: Z = 0.79 (P = 0.43)								
**1.10.2 Riociguat 0.5 to 2.5mg three times daily**								
Ghofrani 2013*	0.6	0.37	151	0.2	4.3	83	14.3%	0.40 [-0.53, 1.33]
Kim 2016	0.8	6.3	105	-0.1	3.6	64	12.5%	0.90 [-0.59, 2.39]
Kim 2016*	0.2	4	46	1	6.3	19	7.6%	-0.80 [-3.86, 2.26]
**Subtotal (95% CI)**			**302**			**166**	**34.5%**	**0.46 [-0.31, 1.22]**
Heterogeneity: Tau² = 0.00; Chi² = 1.00, df = 2 (P = 0.61); I² = 0%								
Test for overall effect: Z = 1.17 (P = 0.24)								
**1.10.3 Riociguat 1.5mg three times daily**								
Dachs 2022	-0.2	6.74	39	-0.4	8.33	48	7.4%	0.20 [-2.97, 3.37]
**Subtotal (95% CI)**			**39**			**48**	**7.4%**	**0.20 [-2.97, 3.37]**
Heterogeneity: Not applicable								
Test for overall effect: Z = 0.12 (P = 0.90)								
**1.10.4 Riociguat 1mg three times daily**								
Bonderman 2013	0.3	2.19	28	-2.7	1.5	56	14.4%	3.00 [2.10, 3.90]
**Subtotal (95% CI)**			**28**			**56**	**14.4%**	**3.00 [2.10, 3.90]**
Heterogeneity: Not applicable								
Test for overall effect: Z = 6.52 (P < 0.00001)								
**1.10.5 Riociguat 2mg three times daily**								
Bonderman 2013*	-4	1.55	54	-2.7	1.5	56	15.2%	-1.30 [-1.87, -0.73]
**Subtotal (95% CI)**			**54**			**56**	**15.2%**	**-1.30 [-1.87, -0.73]**
Heterogeneity: Not applicable								
Test for overall effect: Z = 4.47 (P < 0.00001)								
**Total (95% CI)**			**730**			**508**	**100.0%**	**0.43 [-0.75, 1.61]**
Heterogeneity: Tau² = 2.31; Chi² = 65.52, df = 7 (P < 0.00001); I² = 89%								
Test for overall effect: Z = 0.72 (P = 0.47)								
Test for subgroup differences: Chi² = 63.97, df = 4 (P < 0.00001), I² = 93.7%								

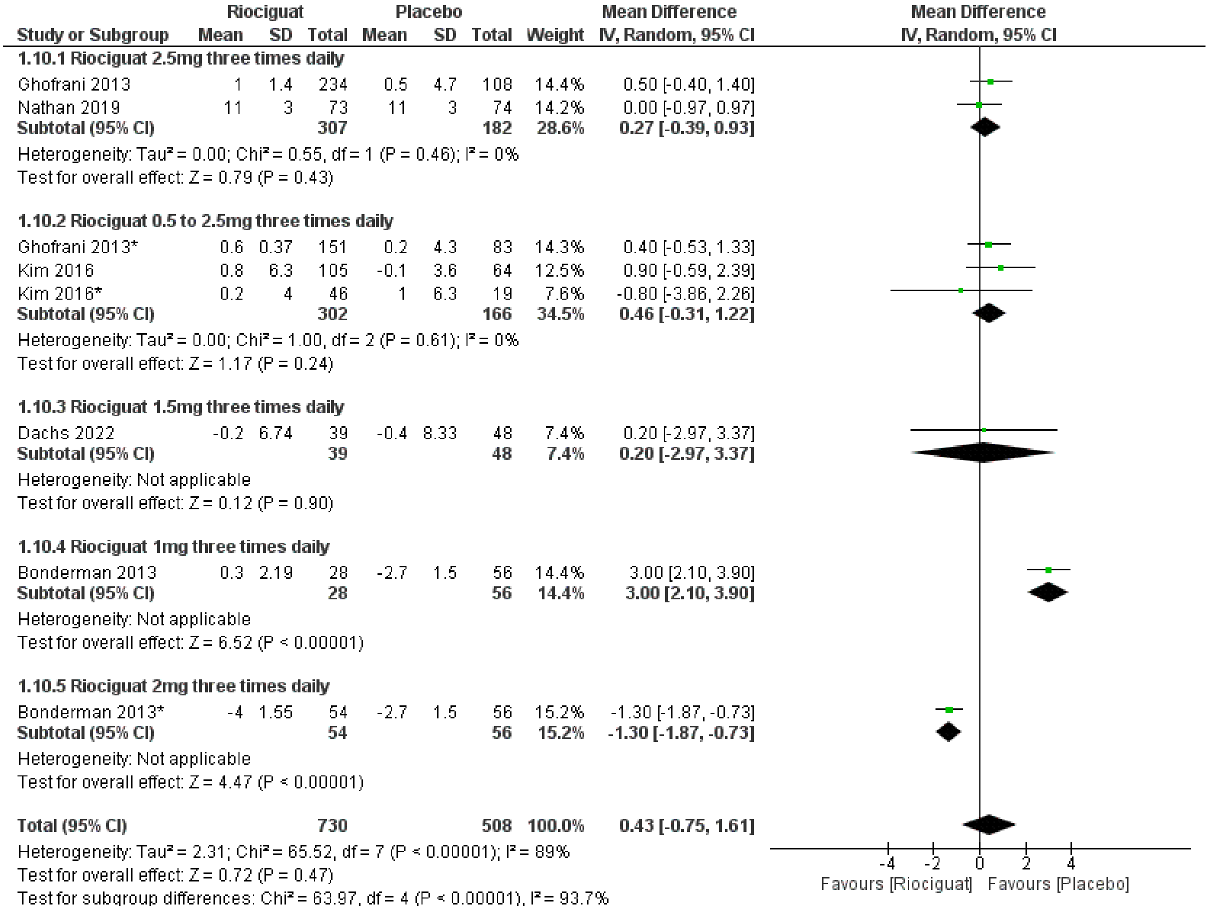

Figures S2 (K): Any Adverse Events

Study or Subgroup	Riociguat Events	Riociguat Total	Placebo Events	Placebo Total	Weight	Risk Ratio M-H, Random, 95% CI
**1.11.1 Riociguat 2.5mg three times daily**						
Galie 2016	107	123	56	66	13.8%	1.03 [0.91, 1.16]
Galie 2016*	120	131	52	60	14.5%	1.06 [0.94, 1.18]
Ghofrani 2013	227	254	108	126	16.3%	1.04 [0.96, 1.13]
Rosenkranz 2015	14	15	10	12	6.0%	1.12 [0.84, 1.49]
**Subtotal (95% CI)**		**523**		**264**	**50.5%**	**1.05 [0.99, 1.11]**
Total events	468		226			
Heterogeneity: Tau² = 0.00; Chi² = 0.36, df = 3 (P = 0.95); I² = 0%						
Test for overall effect: Z = 1.52 (P = 0.13)						
**1.11.2 Riociguate 0.5 to 2.5mg three times daily**						
Ghofrani 2013*	159	173	76	88	15.6%	1.06 [0.97, 1.17]
**Subtotal (95% CI)**		**173**		**88**	**15.6%**	**1.06 [0.97, 1.17]**
Total events	159		76			
Heterogeneity: Not applicable						
Test for overall effect: Z = 1.30 (P = 0.19)						
**1.11.3 Riociguat 1.5mg three times daily**						
Dachs 2022	54	58	55	56	16.6%	0.95 [0.88, 1.03]
Rosenkranz 2015*	6	8	10	12	2.8%	0.90 [0.56, 1.44]
**Subtotal (95% CI)**		**66**		**68**	**19.3%**	**0.95 [0.88, 1.02]**
Total events	60		65			
Heterogeneity: Tau² = 0.00; Chi² = 0.06, df = 1 (P = 0.81); I² = 0%						
Test for overall effect: Z = 1.39 (P = 0.16)						
**1.11.4 Riociguat 1mg three times daily**						
Bonderman 2013	28	85	57	83	4.8%	0.48 [0.34, 0.67]
**Subtotal (95% CI)**		**85**		**83**	**4.8%**	**0.48 [0.34, 0.67]**
Total events	28		57			
Heterogeneity: Not applicable						
Test for overall effect: Z = 4.28 (P < 0.0001)						
**1.11.5 Riociguat 2mg three times daily**						
Bonderman 2013*	61	91	57	83	9.1%	0.98 [0.80, 1.20]
**Subtotal (95% CI)**		**91**		**83**	**9.1%**	**0.98 [0.80, 1.20]**
Total events	61		57			
Heterogeneity: Not applicable						
Test for overall effect: Z = 0.23 (P = 0.82)						
**1.11.6 Riociguat 2mg one time daily**						
Bonderman 2014	5	10	4	13	0.7%	1.63 [0.58, 4.53]
**Subtotal (95% CI)**		**10**		**13**	**0.7%**	**1.63 [0.58, 4.53]**
Total events	5		4			
Heterogeneity: Not applicable						
Test for overall effect: Z = 0.93 (P = 0.35)						
**Total (95% CI)**		**948**		**599**	**100.0%**	**0.99 [0.91, 1.08]**
Total events	781		485			
Heterogeneity: Tau² = 0.01; Chi² = 27.62, df = 9 (P = 0.001); I² = 67%						
Test for overall effect: Z = 0.26 (P = 0.80)						
Test for subgroup differences: Chi² = 25.03, df = 5 (P = 0.0001), I² = 80.0%						

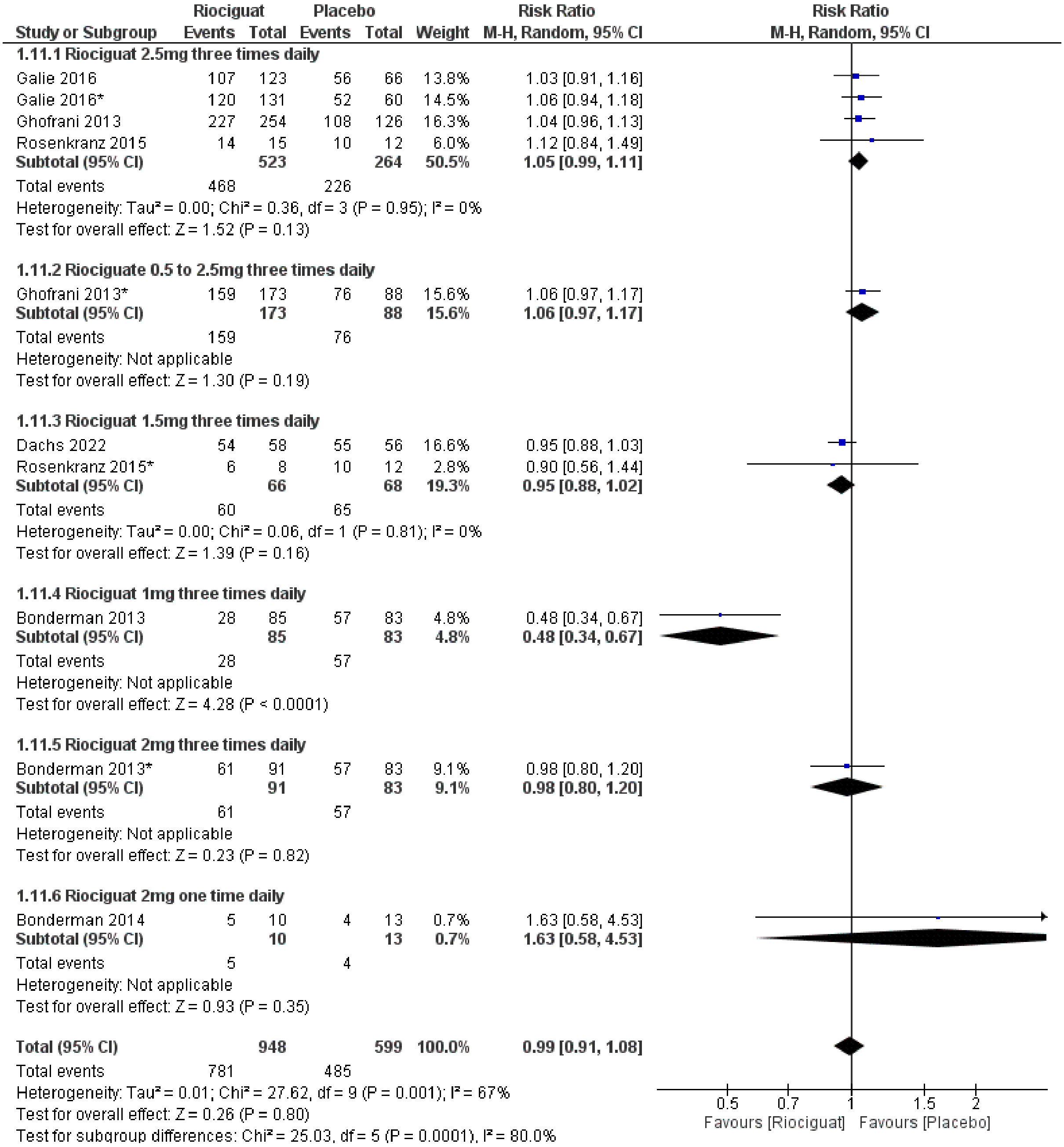

Figures S2 (L): Clinical Worsening Outcome

Study or Subgroup	Riociguat Events	Riociguat Total	Placebo Events	Placebo Total	Weight	Risk Ratio M-H, Random, 95% CI
**1.12.1 Riociguat 2.5mg three times daily**						
Ghofrani 2013	3	254	8	126	42.7%	0.19 [0.05, 0.69]
**Subtotal (95% CI)**		**254**		**126**	**42.7%**	**0.19 [0.05, 0.69]**
Total events	3		8			
Heterogeneity: Not applicable						
Test for overall effect: Z = 2.52 (P = 0.01)						
**1.12.2 Riociguat 0.5 to 2.5mg three times daily**						
Ghofrani 2013*	4	173	5	88	43.3%	0.41 [0.11, 1.48]
**Subtotal (95% CI)**		**173**		**88**	**43.3%**	**0.41 [0.11, 1.48]**
Total events	4		5			
Heterogeneity: Not applicable						
Test for overall effect: Z = 1.37 (P = 0.17)						
**1.12.3 Riociguat 1.5mg three times daily**						
Rosenkranz 2015*	1	8	0	12	13.9%	4.33 [0.20, 94.83]
**Subtotal (95% CI)**		**8**		**12**	**13.9%**	**4.33 [0.20, 94.83]**
Total events	1		0			
Heterogeneity: Not applicable						
Test for overall effect: Z = 0.93 (P = 0.35)						
**Total (95% CI)**		**435**		**226**	**100.0%**	**0.40 [0.11, 1.44]**
Total events	8		13			
Heterogeneity: Tau² = 0.53; Chi² = 3.51, df = 2 (P = 0.17); I² = 43%						
Test for overall effect: Z = 1.40 (P = 0.16)						
Test for subgroup differences: Chi² = 3.50, df = 2 (P = 0.17), I² = 42.9%						

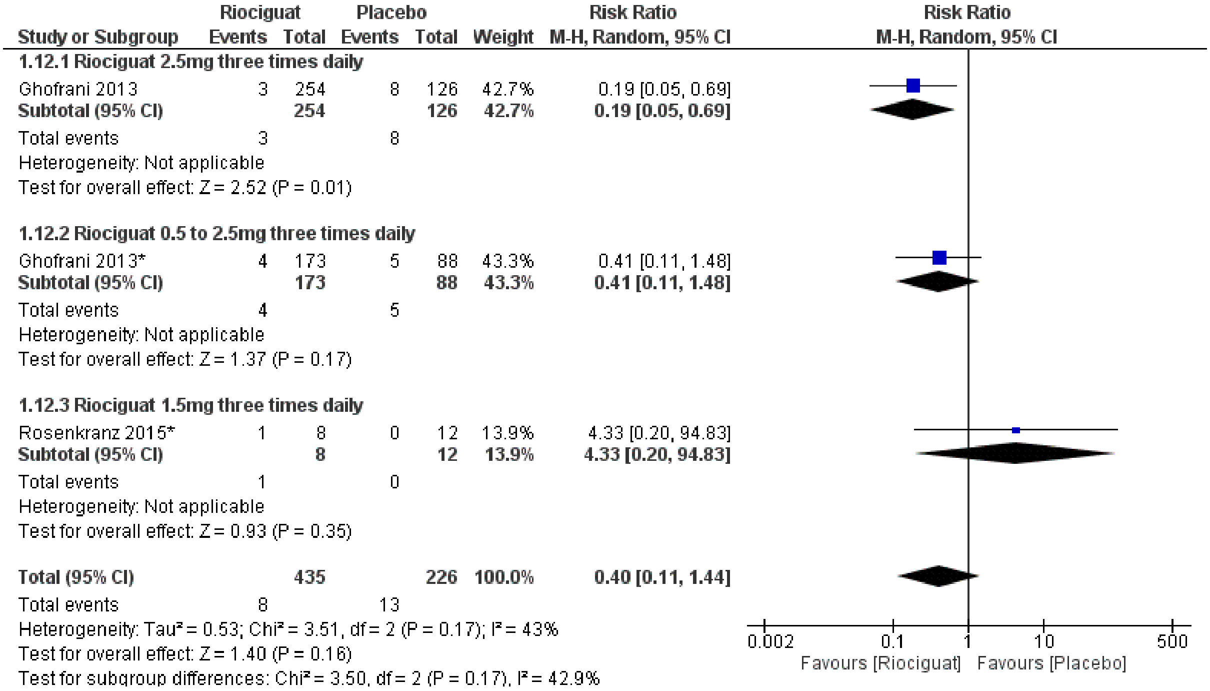

Supplementary Digital Material: Funnel Plot

Figure S3 (A): Six-minute Walk distance (6MWD)

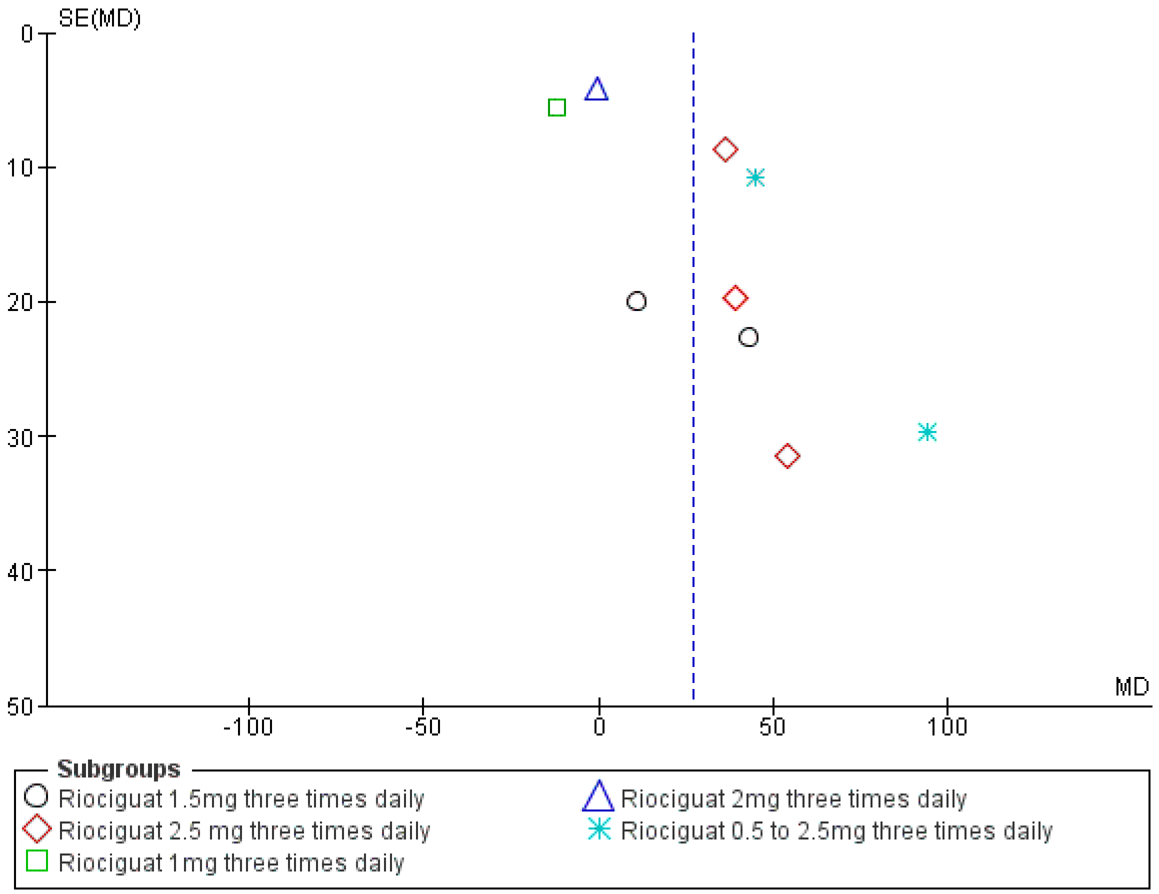

Supplementary Digital Material: Funnel Plot

Figure S3 (B): Mean Pulmonary Artery Pressure (mPAP)

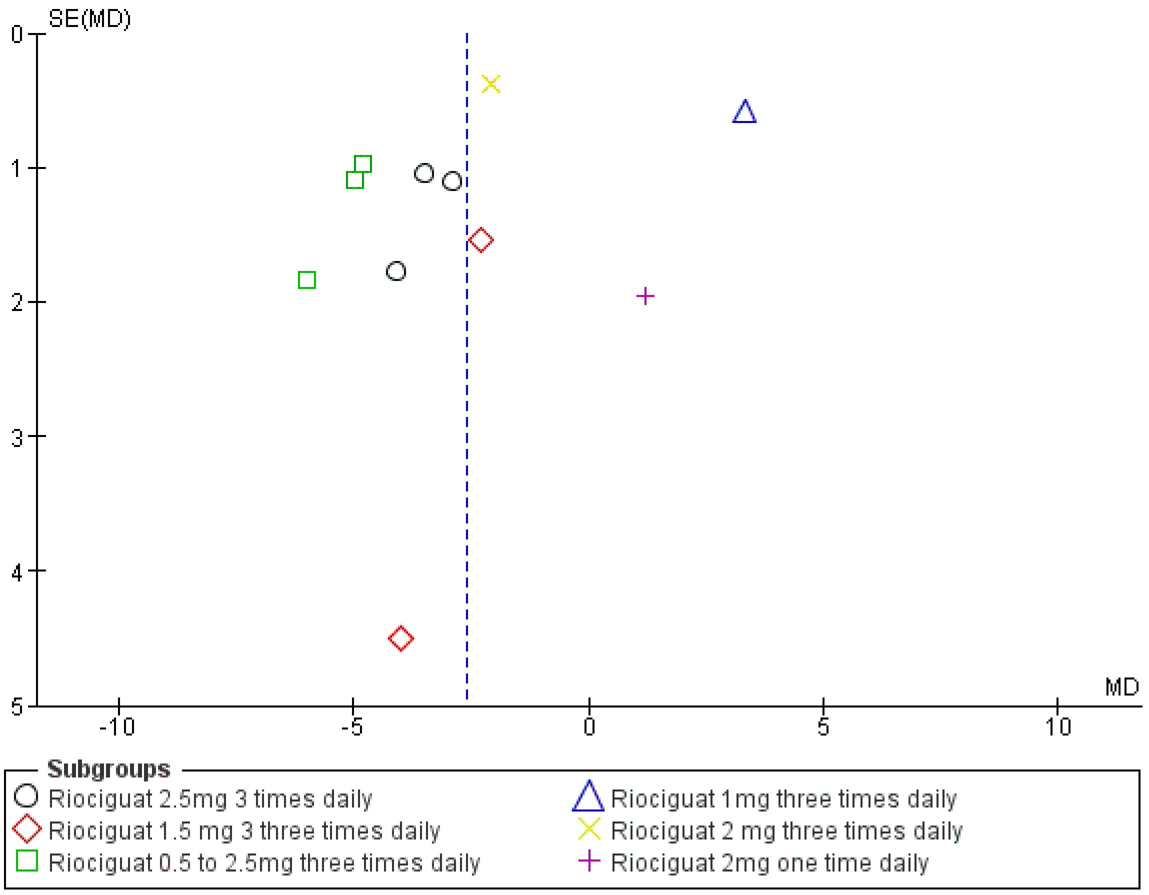

Supplementary Digital Material: Funnel Plot

Figure S3 (C): Pulmonary Vascular Resistance

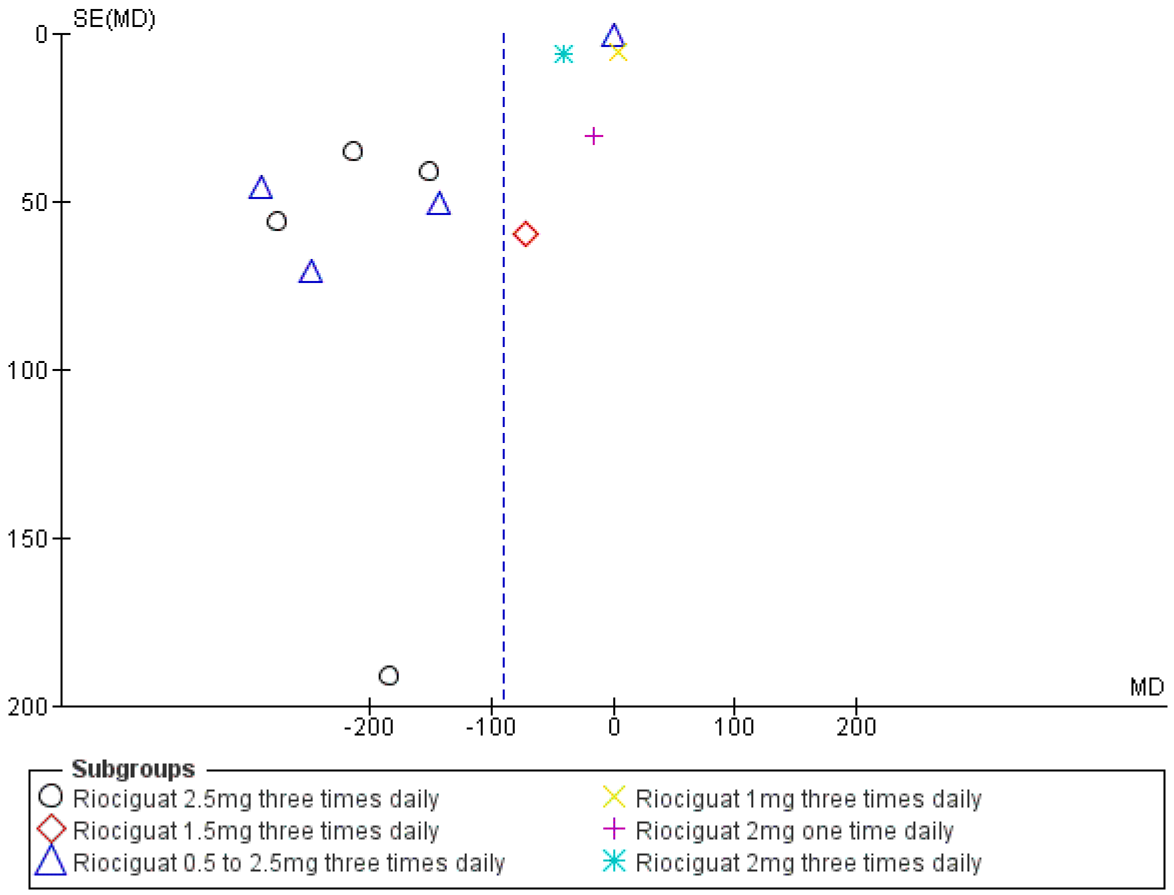

Supplementary Digital Material: Funnel Plot

Figure S3 (D): Right Atrial Pressure (RAP)

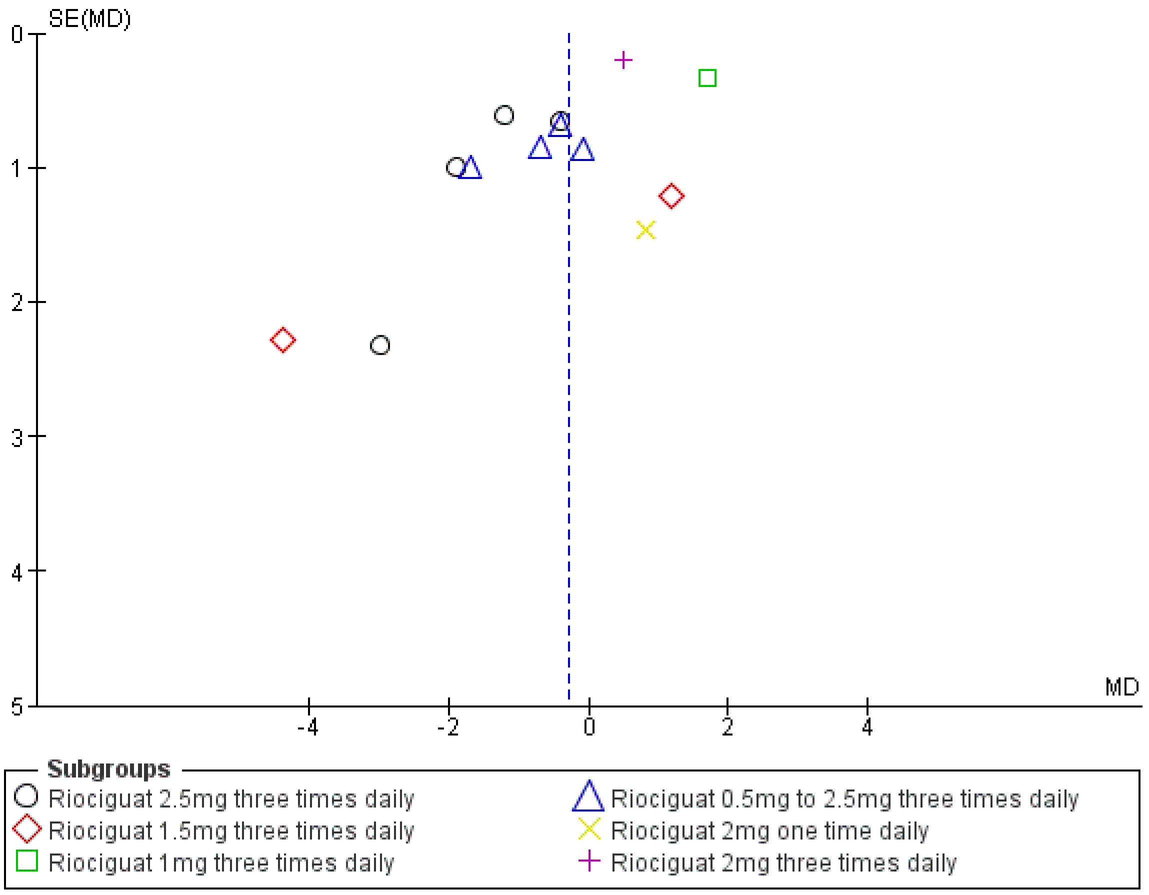

Supplementary Digital Material: Funnel Plot

Figure S3 (E): Mean Arterial Pressure (MAP)

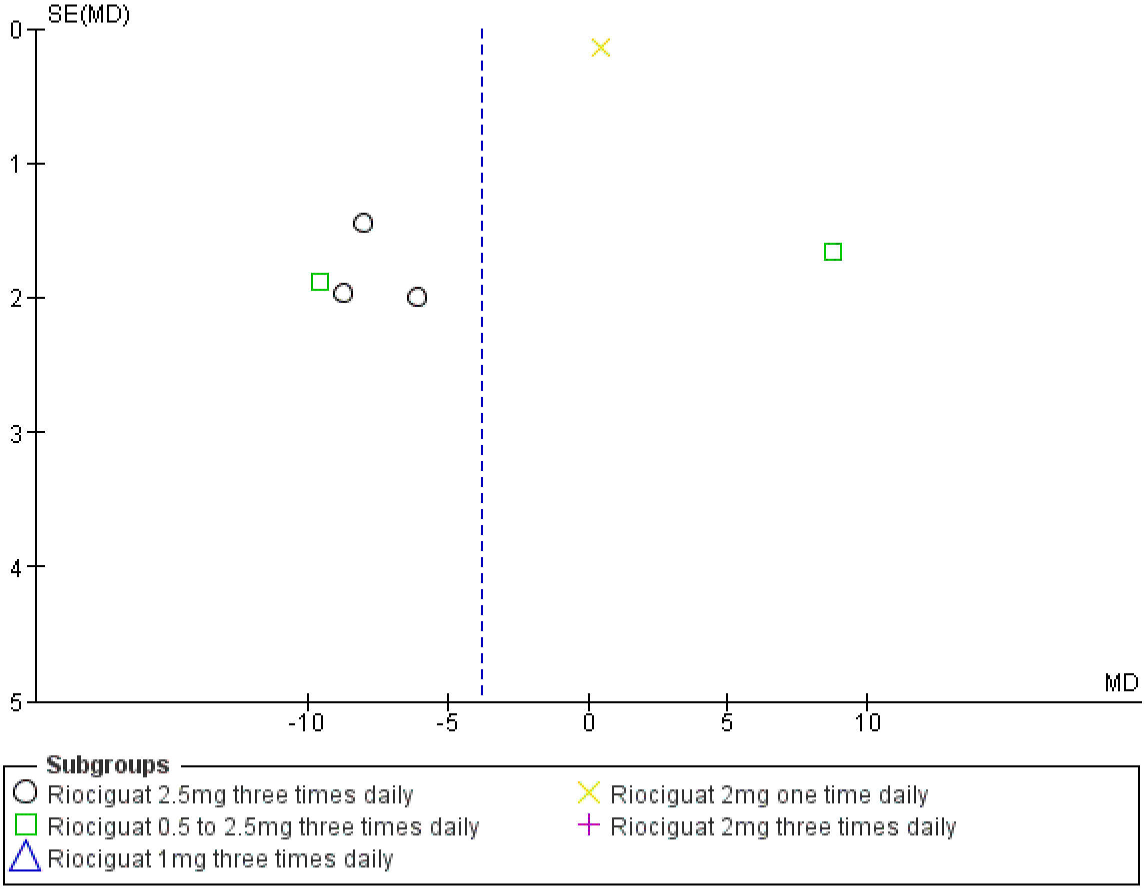

Supplementary Digital Material: Funnel Plot

Figure S3 (F): Cardiac Index

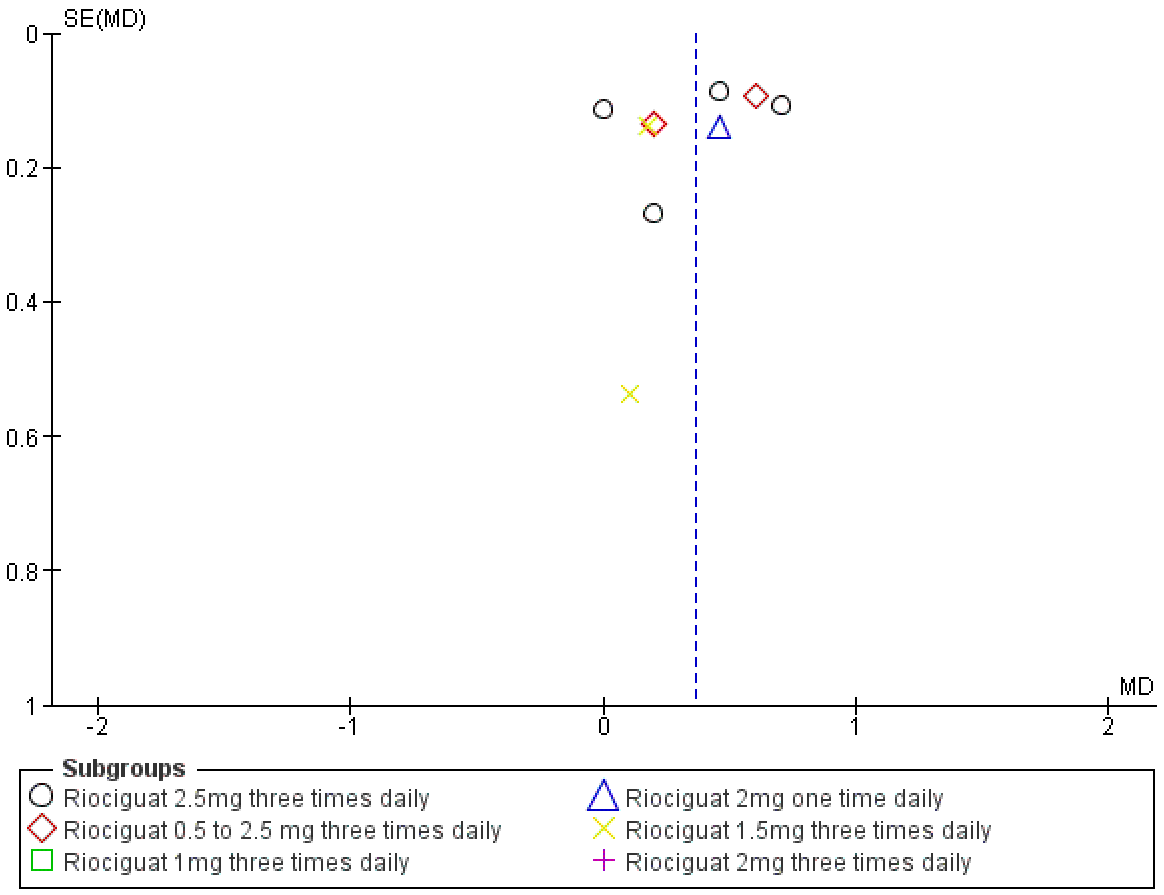

Supplementary Digital Material: Funnel Plot

Figure S3 (G): Cardiac Output

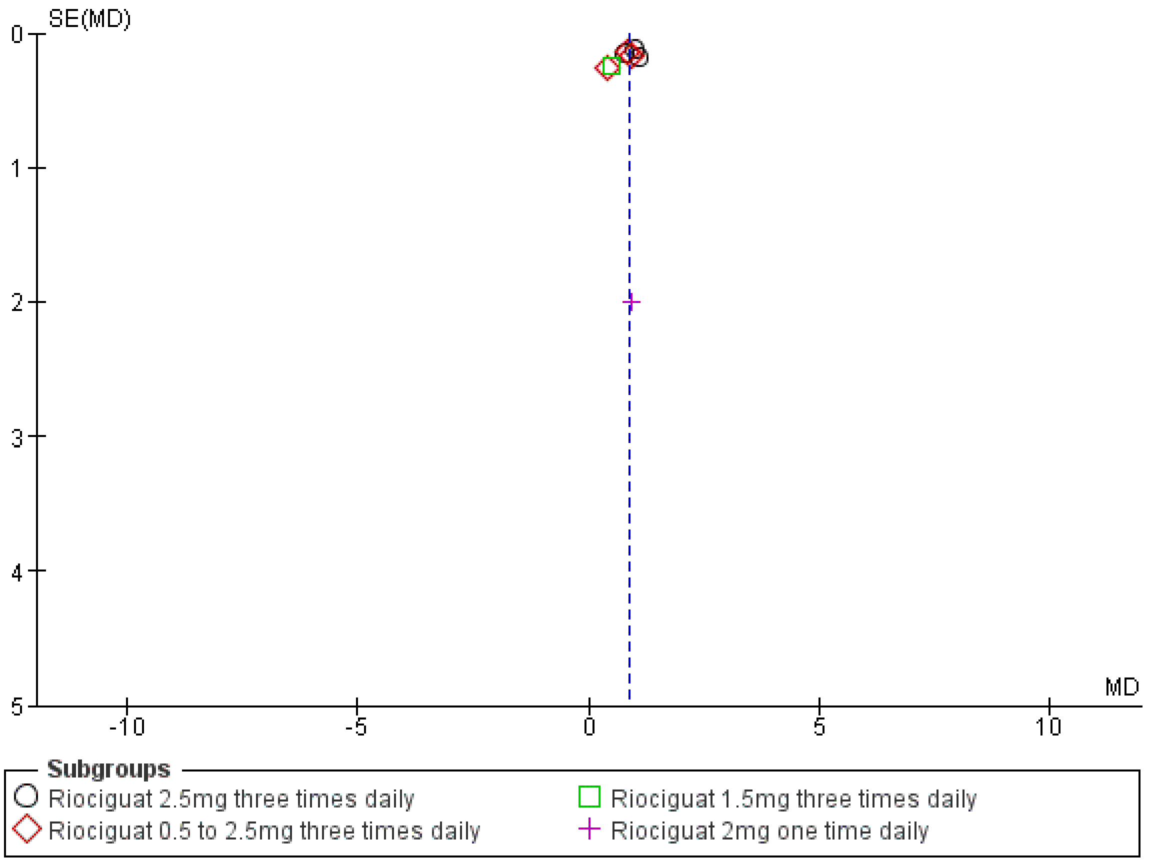

Supplementary Digital Material: Funnel Plot

Figure S3 (H): N-terminal pro-type B natriuretic peptide (NT-proBNP)

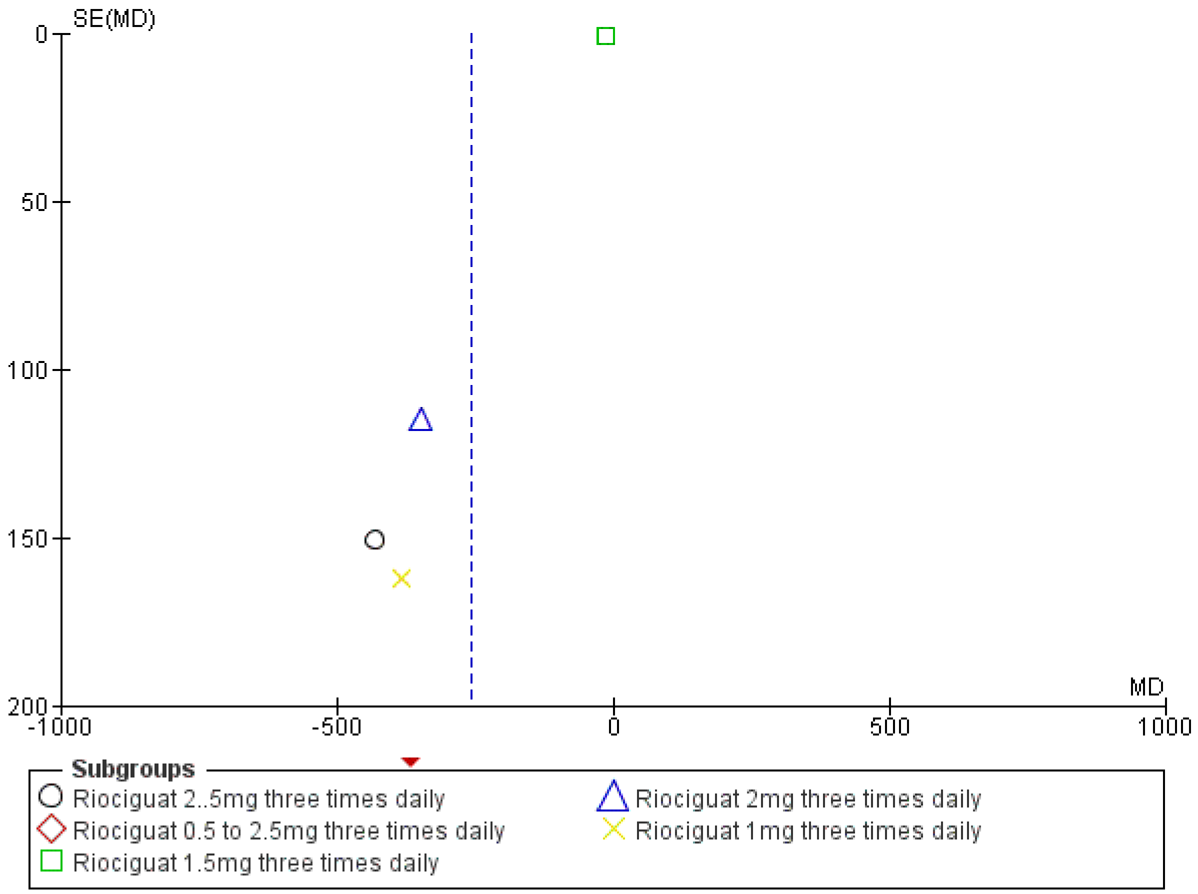

Supplementary Digital Material: Funnel Plot

Figure S3 (I): Systemic Vascular Resistance

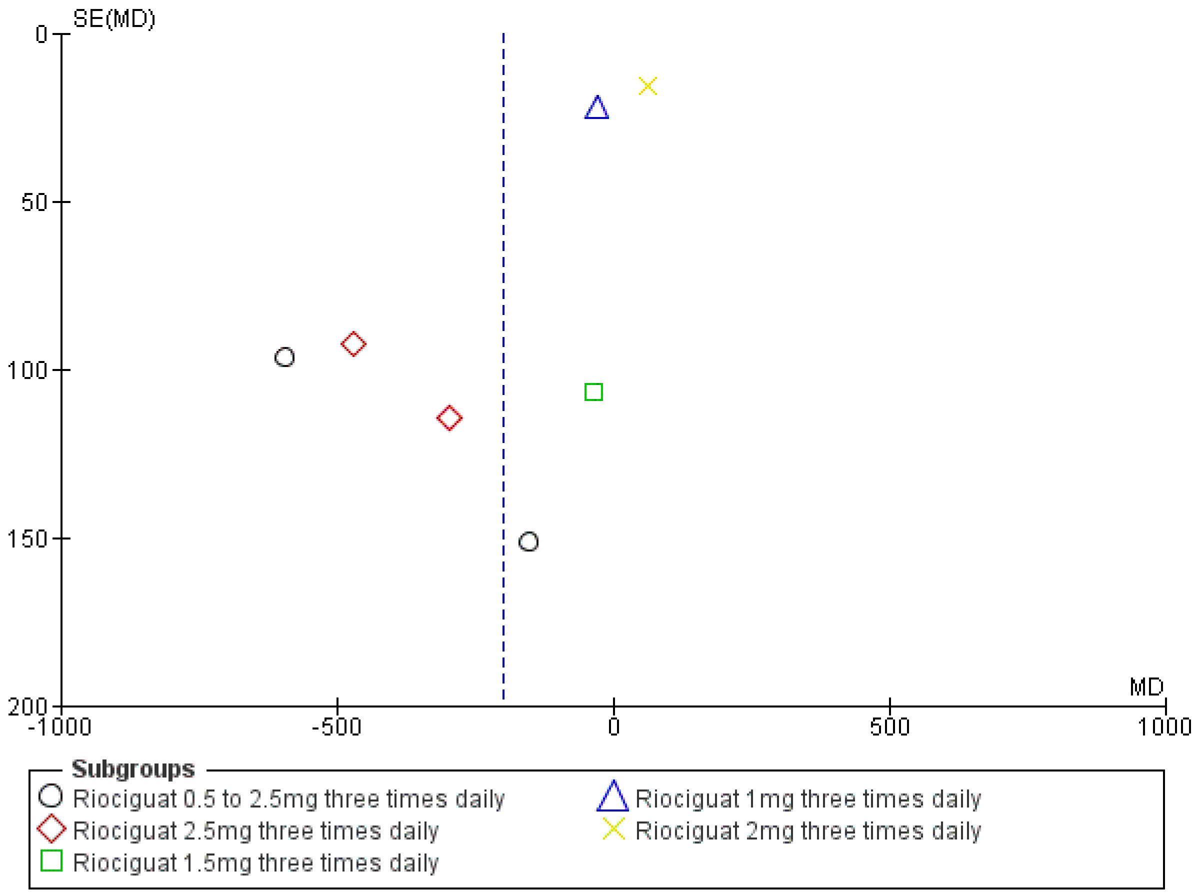

Supplementary Digital Material: Funnel Plot

Figure S3 (J): Pulmonary Artery Wedge Pressure

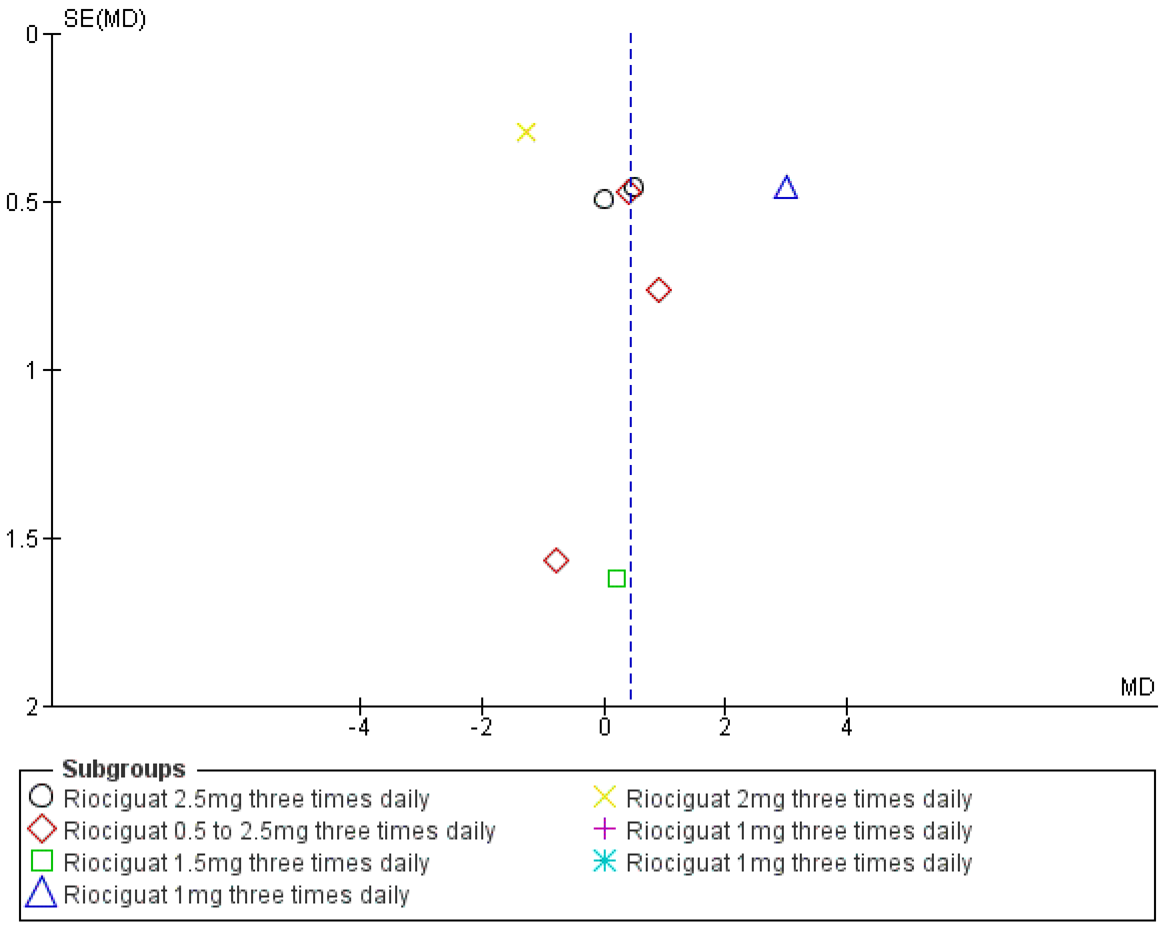

Supplementary Digital Material: Funnel Plot

Figure S3 (K): Any Adverse Events

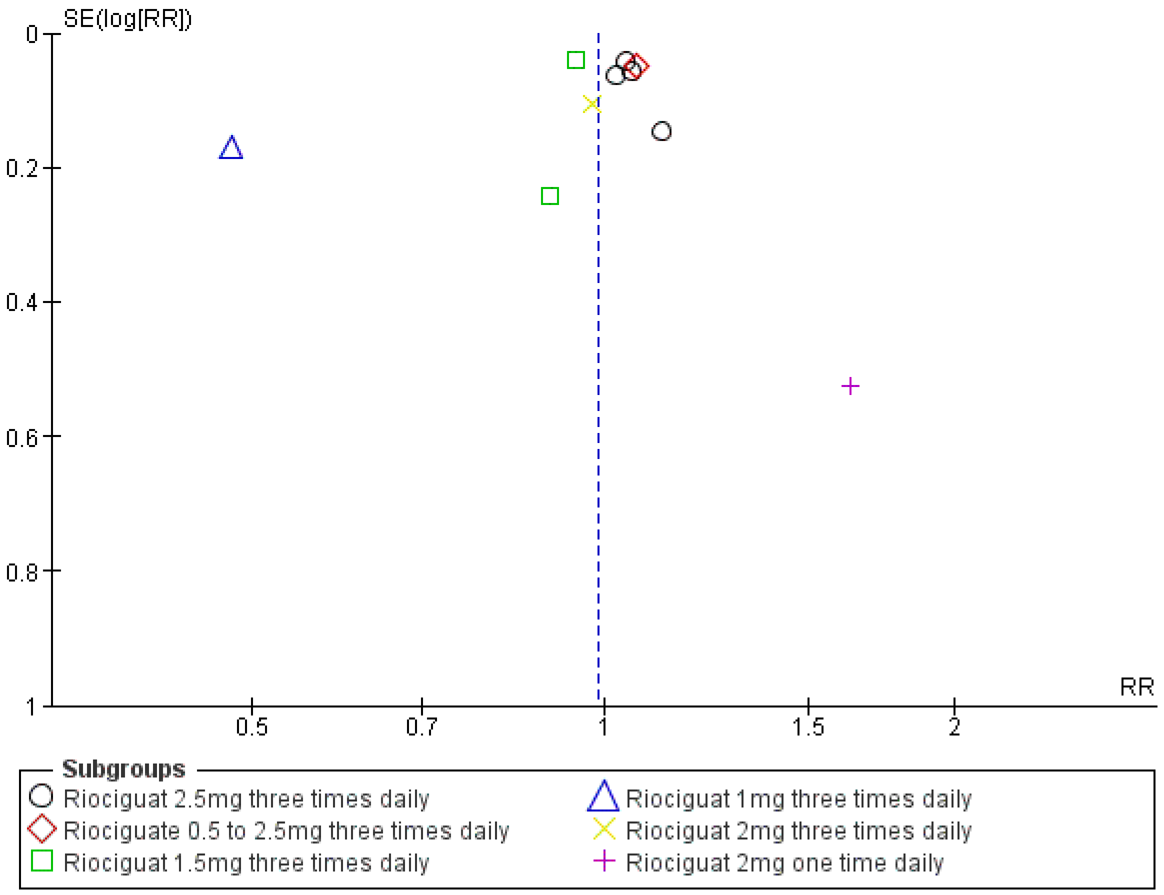

Supplementary Digital Material: Funnel Plot

Figure S3 (L): Clinical Worsening Outcome

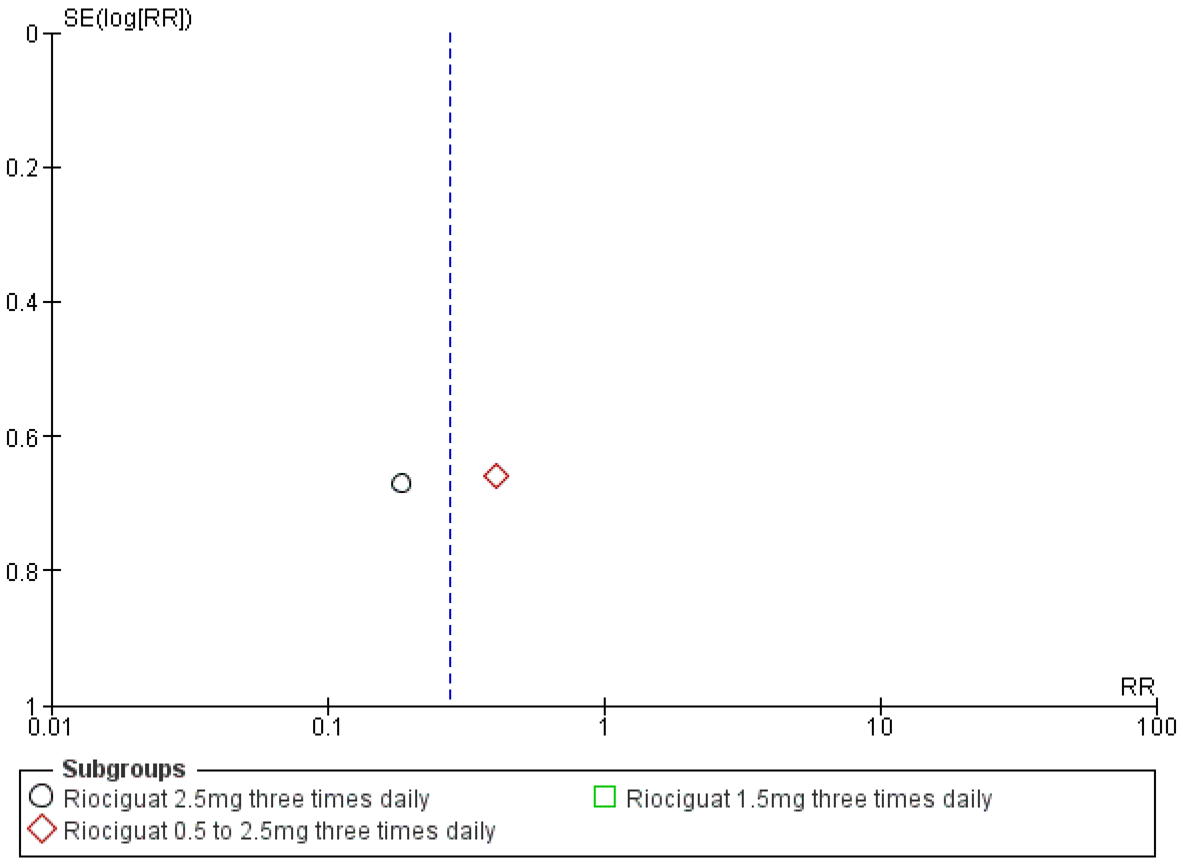

Table S1, Supplementary Digital Material: Search String Table

Databases	Search Strings
MEDLINE	"((((riociguat) OR (BAY 63-2521)) OR (BAY-63-2521)) OR (Adempas)) AND (((((((chronic thromboembolic pulmonary hypertension) OR (chronic pulmonary embolism)) OR (CTEPH)) OR (pulmonary arterial hypertension)) OR (PAH)) OR (pulmonary hypertension)) OR (PH))”
CENTRAL	“ ((((riociguat) OR (BAY 63-2521)) OR (BAY-63-2521)) OR (Adempas)) AND (((((((chronic thromboembolic pulmonary hypertension) OR (chronic pulmonary embolism)) OR (CTEPH)) OR (pulmonary arterial hypertension)) OR (PAH)) OR (pulmonary hypertension)) OR (PH)) “
Google Scholar	“ ((((riociguat) OR (BAY 63-2521)) OR (BAY-63-2521)) OR (Adempas)) AND (((((((chronic thromboembolic pulmonary hypertension) OR (chronic pulmonary embolism)) OR (CTEPH)) OR (pulmonary arterial hypertension)) OR (PAH)) OR (pulmonary hypertension)) OR (PH)) “
Embase	“ ((((riociguat) OR (BAY 63-2521)) OR (BAY-63-2521)) OR (Adempas)) AND (((((((chronic thromboembolic pulmonary hypertension) OR (chronic pulmonary embolism)) OR (CTEPH)) OR (pulmonary arterial hypertension)) OR (PAH)) OR (pulmonary hypertension)) OR (PH)) “
